# Subunit vaccine protects against a clinical isolate of *Mycobacterium avium* in wild type and immunocompromised mouse models

**DOI:** 10.1038/s41598-021-88291-8

**Published:** 2021-04-27

**Authors:** Sasha E. Larsen, Valerie A. Reese, Tiffany Pecor, Bryan J. Berube, Sarah K. Cooper, Guy Brewer, Diane Ordway, Marcela Henao-Tamayo, Brendan K. Podell, Susan L. Baldwin, Rhea N. Coler

**Affiliations:** 1 Center for Global Infectious Disease Research, Seattle Children’s Research Institute, Seattle, WA USA; 2 Department of Microbiology, Immunology and Pathology, Colorado State University, Fort Collins, CO USA; 3 Alternative Behavior Strategies Inc, Salt Lake City, UT USA

**Keywords:** Optical imaging, Mechanisms of disease, Vaccines, Adaptive immunity, Imaging the immune system, Immunotherapy, Infection, Infectious diseases, Translational immunology, Vaccines, Applied microbiology, Bacteriology, Pathogens, Vaccines

## Abstract

The nontuberculous mycobacteria (NTM) *Mycobacterium avium* is a clinically significant pathogen that can cause a wide range of maladies, including tuberculosis-like pulmonary disease. An immunocompromised host status, either genetically or acutely acquired, presents a large risk for progressive NTM infections. Due to this quietly emerging health threat, we evaluated the ability of a recombinant fusion protein ID91 combined with GLA-SE [glucopyranosyl lipid adjuvant, a toll like receptor 4 agonist formulated in an oil-in-water stable nano-emulsion] to confer protection in both C57BL/6 (wild type) and Beige (immunocompromised) mouse models. We optimized an aerosol challenge model using a clinical NTM isolate: *M. avium* 2-151 smt, observed bacterial growth kinetics, colony morphology, drug sensitivity and histopathology, characterized the influx of pulmonary immune cells, and confirmed the immunogenicity of ID91 in both mouse models. To determine prophylactic vaccine efficacy against this *M. avium* isolate, mice were immunized with either ID91 + GLA-SE or bacillus Calmette–Guérin (BCG)*.* Immunocompromised Beige mice displayed a delayed influx of innate and adaptive immune cells resulting in a sustained and increased bacterial burden in the lungs and spleen compared to C57BL/6 mice. Importantly, both ID91 + GLA-SE and BCG vaccines significantly reduced pulmonary bacterial burden in both mouse strains. This work is a proof-of-concept study of subunit vaccine-induced protection against NTM.

## Introduction

Nontuberculous mycobacteria (NTM) comprising 170 different species [and excluding *Mycobacterium tuberculosis* (Mtb) and *M. leprae*] are an emerging global health concern. Despite garnering less attention than Mtb, NTM cause a significant amount of morbidity, mortality and financial health burden in certain regions of the world, including the United States^1^. Indeed, exposure to NTM measured by skin sensitization testing reveals a steady increase (up to 16.6% sensitivity to *M. intracellulare* in 2000), with a corresponding decrease in skin sensitization to Mtb (down to 5.6% in 2000) in the United States over the same period tested^2^. Using death certificate reporting, researchers calculated that the age adjusted mortality rate in the United States for Mtb was 3.3 per million person years and 2.3 per million person years for NTM and rising annually in individuals not living with HIV^3^. These inverse trends between mycobacteria are reflected globally^4^. A systematic review revealed that NTM infection rates were increasing across 75% of geographic areas evaluated with 81% demonstrating declines in tuberculosis (TB) incidence^5^. Susceptibility to different NTM species, including *M. avium*, is influenced by complex host factors and the extent of pathogen exposure. The wide range of illness and disease disproportionately affects immune compromised individuals, and a suppressed immune system, either genetic or acquired, is a prominent risk factor for opportunistic NTM infections^6–8^.

NTM species are ubiquitous in the environment and can be found in soil, water or residing on fomites, which can result in repetitive host exposure^9,10^. Therefore, it is not surprising that the incidence of mycobacterial disease caused by NTM infections have steadily increased globally over the last 60 years^11^. Predominant NTM species that cause significant pulmonary disease include *Mycobacterium avium complex* (MAC), *M. kansasii* and *M. abscessus*^12^*.* From 2009 to 2013 approximately 61–91% of patients evaluated for pathogenic NTM infection in the United States were confirmed to have MAC, while *M. abscessus* and *M. kansasii* ranged from 2 to 18%^13^.

Despite an increased awareness of NTM infections, true incidence is likely underreported^14–16^. NTM disease manifestation can be remarkably similar to TB including chronic cough, lesions on chest X-ray and progressive fatigue^17^, resulting in misdiagnosis. Indeed, 18% of ‘chronic TB cases’ in Mali were identified as NTM positive upon reevaluation by microscopy, culture and drug sensitivity testing^18^. Unfortunately, those patients misdiagnosed as having active Mtb infections are usually given treatments for MDR-TB. Even if properly identified, MAC is a non-reportable infectious disease in North America, which leads to great variance of incidence estimates. Centers for Disease Control and Prevention surveillance data suggests an incidence rate of 1 case per 100 k person-years in the United States. A separate study of the United States Health Administration observed an incidence of NTM infection of 12.6 cases per 100 k person-years in the veteran population^19^, suggesting a disproportionate disease burden in certain communities exist for NTM. Due to increasing prevalence, insidious virulence, and accelerating drug resistance, NTM disease is an underappreciated but growing public health burden.

Proper identification does not ensure positive prognosis, as NTM infections are notoriously difficult to treat. Current diagnostic methods are severely inadequate and clinical disease is often mistreated with ineffective drugs^20,21^. Even when correctly diagnosed, a multidrug therapy is necessary for NTM treatment with contraindications for certain at risk populations^22^. A regimen of rifampin, ethambutol and clarithromycin or azithromycin for a period of at least 12 months following sputum culture conversion is the current recommendation for treating pulmonary MAC infection^23^. Historically, a meager 50–70% of individuals undergoing the correct complex multi-drug treatment regimen for chronic MAC infections reach cure^24^ and even then some treated patients experience relapse infections or recurrent infections with different strains of mycobacteria^20^. In addition, there is increasing evidence of NTM strains that are refractory to antibiotic treatment^20,25,26^. Novel drug therapies against MAC are being investigated for safety and efficacy^23,27^, but use of these new drugs may also eventually lead to the emergence of drug resistance and recurrence of disease.

The growing incidence of NTM infection combined with misdiagnosis and common treatment failure represent an expanding unmet medical need^17^. An alternative or complementary approach for improved therapies against NTM disease is to design, develop and evaluate vaccines against NTM. Vaccine strategies against NTM may help reduce incidence of disease and/or recurrence and could increase patient compliance by reducing the necessary duration of antibiotic treatment. Toxicity is well documented with prolonged drug regimens against NTM infections^28^, and therefore a shortened regimen of treatment afforded by vaccines would be of large benefit to those affected.

In this work we aimed to advance and characterize a reproducible preclinical pulmonary challenge model of a virulent strain of *M. avium* subspecies hominissuis in mice*.* This enabled immunogenicity and efficacy assessment using a proof-of-concept subunit vaccine candidate against *M. avium* infection in both wild type (C57BL/6) and immunocompromised (Beige) preclinical mouse models. Although a T helper 1 (T_H_1) immune response is partially essential for resistance to NTM infections, complete and reliable immune correlates of protection have not yet been established^12^. Therefore, we surveyed vaccine-induced responses that correlated with efficacy. These data are evidence that subunit vaccination against NTM species, including *M. avium*, is a viable mechanism to reduce this disease burden.

## Results

### *M. avium* 2-151 smt productively infects C57BL/6 mice

**Figure 1:**
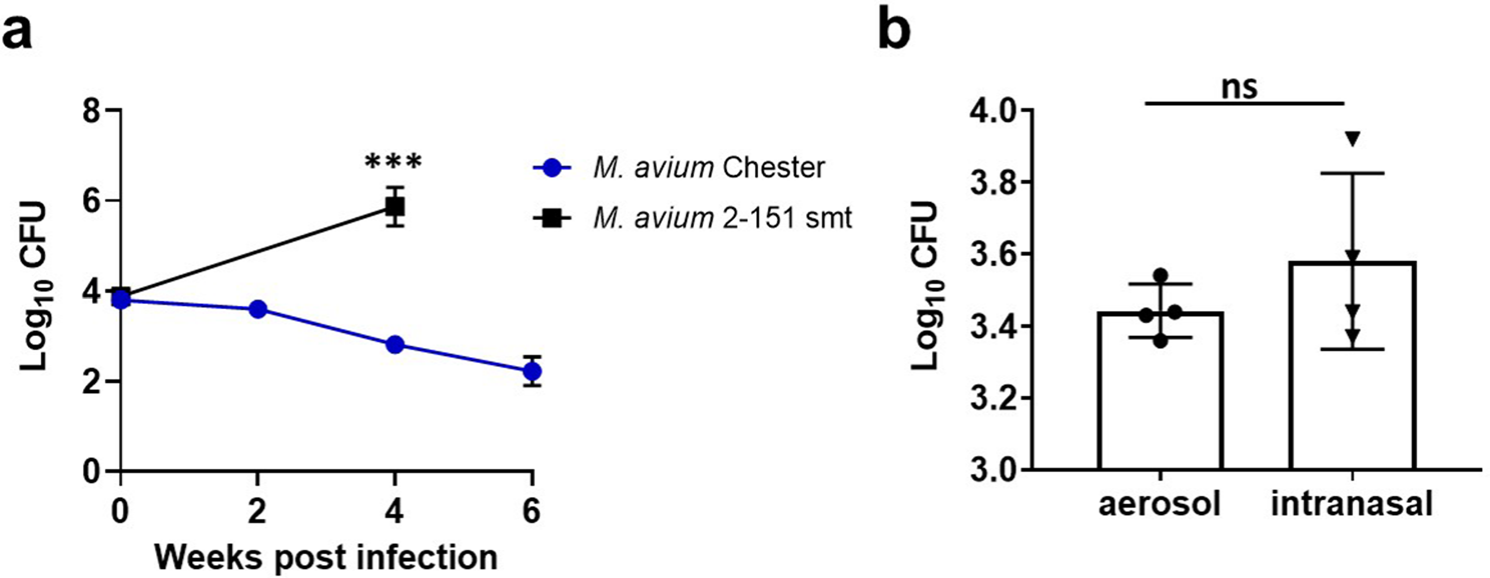
*M. avium* 2-151 smt establishes a persistent infection in C57BL/6 mice and is effectively delivered via aerosol. (**a**) Lung bacterial burden assessed by colony forming units (CFU) 24 h and 2, 4 and 6 weeks post intranasal infection with *M. avium* Chester or *M. avium* 2-151 smt in C57BL/6 mice, n = 3–7 mice per time point, data represents mean ± SD. (**b**) Lung bacterial burden assessed by CFU 24 h post infection by aerosol or intranasal infection, n = 4 mice per group.

In order to assess the efficacy of vaccination and subsequent immune responses against NTM, we first established viable mouse models of disease. Since NTM infection and disease burden are more pronounced in immunocompromised populations, our investigation included the Beige mouse strain as the representative immunocompromised mouse model and the C57BL/6 mouse strain as a wild type (WT) resistant mouse strain. Indeed, Beige and C57BL/6 mice have been leveraged for comparative assessments of *M. avium* infections before, utilizing intranasal infections to evaluate the relative contributions of innate immune cells during early infection^29^. Other publications have used C57BL/6 mice with intratracheal installation^30^ as well as intraperitoneal injection^31^ to compare virulence and pathogenesis of *M. avium* isolates. A review of models for NTM therapies can be found here^32^. In this work we aim to expand these models using virulent *M. avium* isolates and aerosol infection routes, which we and others have used previously for mycobacterial challenge^33,34^, to evaluate vaccine-induced immunity. Interestingly, Beige mice demonstrate enhanced susceptibility to mycobacterial infections due to reduced bactericidal activity of granulocytes, a deficiency in NK cells, and a reported minor defect/delay in humoral responses^35–37^. We utilized the clinical isolate *M. avium 2-151* smooth transparent (smt) as a challenge strain (generously provided by D. Ordway, Colorado State University) due to its enhanced virulence compared to the commonly used *M. avium* Chester laboratory-adapted strain, and its ability to establish a persistent pulmonary infection in the mouse model. *M. avium* 2-151 smt infection better represents human disease conditions from NTM infection and provides resolution for deciphering vaccine-induced protection. Indeed, while *M. avium* Chester bacterial burden significantly wanes over time following intranasal challenge, from the starting mean infectious challenge dose of 3.80 Log_10_ colony forming units (CFU) down to a mean of 2.52 Log_10_ CFU six weeks post infection (Fig. 1a), *M. avium* 2-151 smt establishes a sustained infection after intranasal challenge, with an average lung bacterial burden of 5.87 Log_10_ CFU four weeks post challenge, up from the challenge dose (Fig. 1a). This sustained infection of clinical MAC isolates is in agreement with published studies^33,38,39^ across several models as well as in our own data, whereby C57BL/6 mice challenged with aerosol MAC 101, MAC 2285 R or MAC 2285 S^38^ sustain a persistent infection as measured up to 6 weeks post challenge (Supplemental Figure S1). Next, we optimized previous models using a virulent *M. avium* isolate and empirically developed the low dose aerosol (LDA) infection parameters for *M. avium* 2-151 smt (Fig. 1b), making it better aligned with the human aerosol route of infection.

### In vivo growth kinetics and immune responses in C57BL/6 and Beige mice

**Figure 2:**
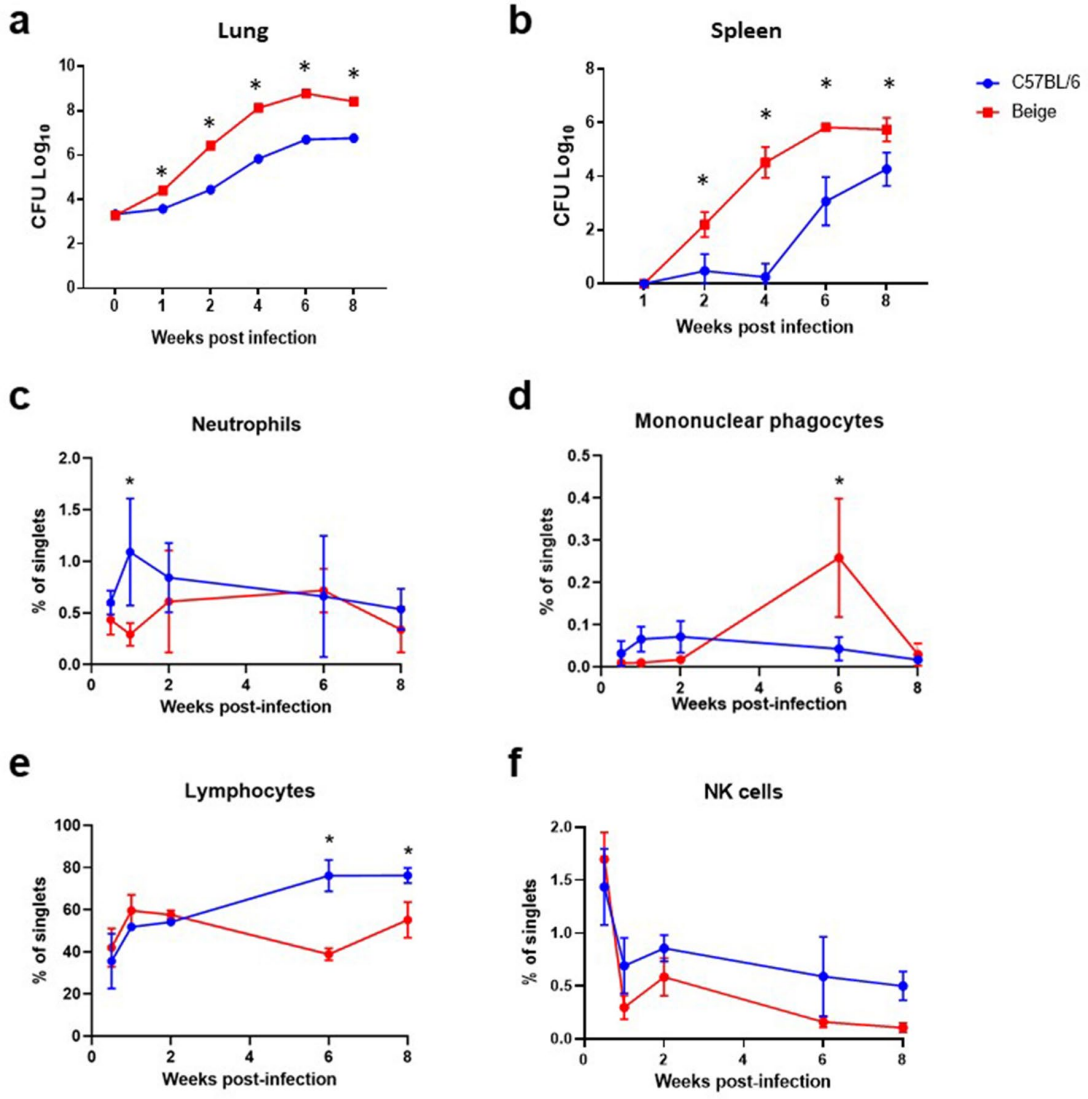
Beige mice sustain a greater bacterial burden of *M. avium* 2-151 smt over time than C57BL/6. Bacterial burden in the (**a**) lung and (**b**) spleen assessed by CFU enumeration weekly post aerosol challenge with *M. avium* 2-151 smt in C57BL/6 (blue circles) and Beige mice (red squares), n = 4 mice per time point. (**c**–**f**) Percent of singlet cells from lung homogenate analyzed 24 h to 8 weeks post infection by flow cytometry for (**c**) neutrophils: Ly6G + Ly6C + , (**d**) mononuclear phagocytes: CD64 + CD11b + , (**e**) Lymphocytes: Ly6G− Ly6C− CD11c− CD11b− and (**f**) NK cells: CD64− NK1.1 + . Mean with SD represented, n = 4 mice per time point. *p < 0.05, unpaired t-test with Holm-Sidak correction for multiple comparisons.

We next evaluated the in vivo growth kinetics of *M. avium* 2-151 smt in both the C57BL/6 and Beige models to determine the host-induced immune response to infection, histopathology as well as potential bacterial phenotypic differences between the WT and immunocompromised hosts. Lungs and spleens were harvested and homogenates were plated 24 h, 1, 2, 4, 6 and 8 weeks post infection to determine bacterial burden and colony phenotypes. Not surprisingly, despite receiving the same challenge dose, Beige mice were less able to control the infection with the clinical *M. avium* isolate, resulting in significantly higher bacterial counts in the lung and spleen over time, from one to eight weeks post challenge (Fig. 2a,b). This accelerated increase in bacterial burden in Beige mice corresponds with a two-week delay in neutrophil and mononuclear phagocyte pulmonary influx (Fig. 2c,d) and significantly decreased percentage of pulmonary lymphocytes (Fig. 2e, Supplemental Figure S2) 6–8 weeks post infection when compared to C57BL/6 mice. Indeed, previous reports have demonstrated that a decreased neutrophil recruitment in Beige mice is in part responsible for enhanced susceptibility to intranasal challenge with *M. avium* when compared to C57BL/6 mice^40,41^. Conversely, NK cell depletion in either mouse model has historically not influenced susceptibility to *M. avium* infection^40^. We confirmed in our model of *M. avium* 2-151 smt aerosol challenge that the kinetics and magnitude of pulmonary NK cells was not significantly different between Beige and C57BL/6 mice out to 8 weeks post infection (Fig. 2f).

**Figure 3:**
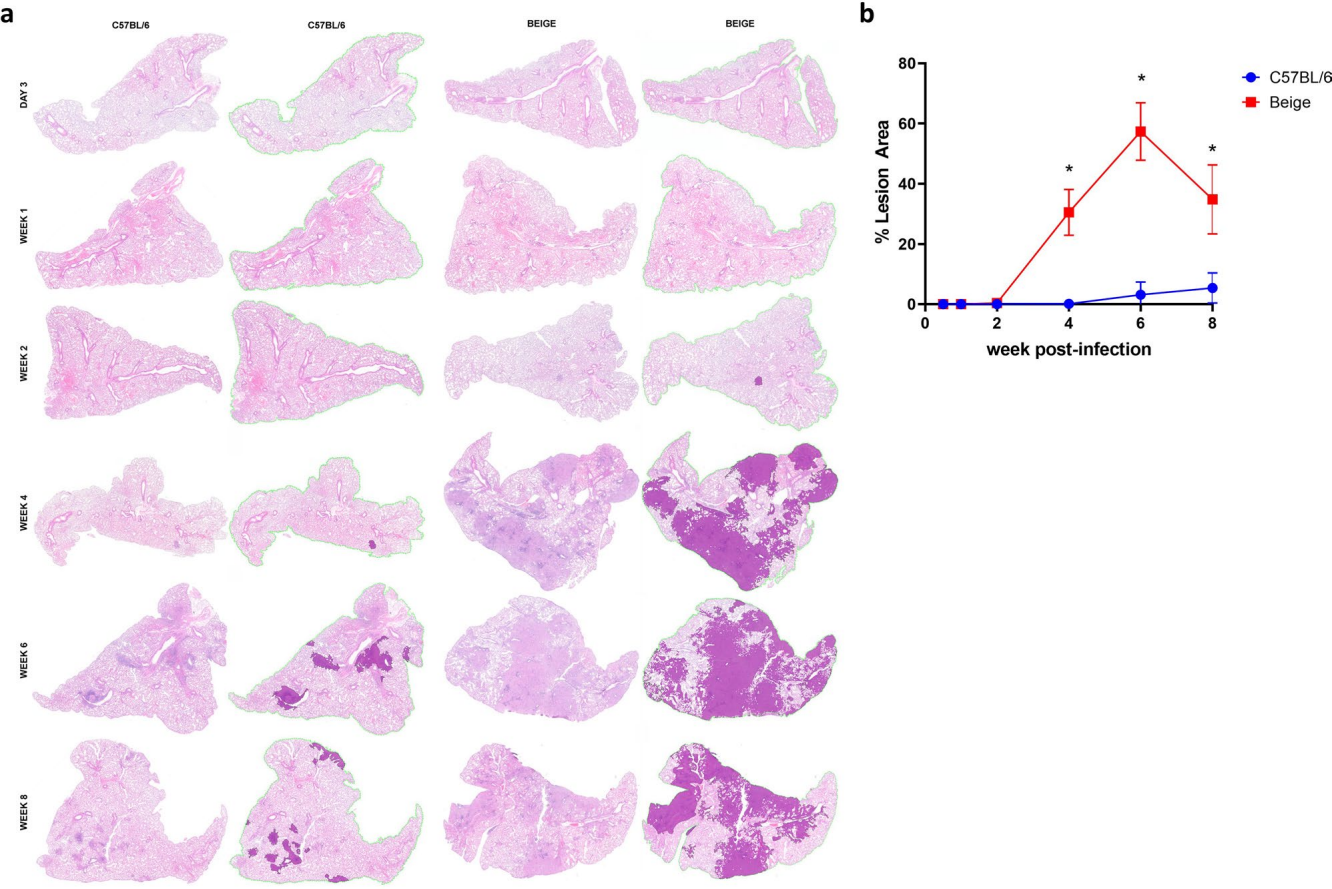
Kinetic quantitative histopathology using a novel high-throughput algorithm. (**a**) Representative accessory lung lobes from C57BL/6 (Group 1) and Beige (Group 2), before (left column) and after (right column) algorithmic scoring of H&E staining for pulmonary lesions (dark purple). (**b**) Mean ± SD percent lesion area, n = 3–4 animals per group per time point. *p < 0.05, Two-Way ANOVA with Sidak’s multiple comparison test between mouse strains at each time point.

In addition to flow cytometric analysis of the kinetic influx of immune cells to the lung we also evaluated pulmonary immunopathology over the course of infection using Visiopharm software and a novel, custom-made algorithm using decision forest training and classification (at Colorado State University) to detect areas of inflammation, or lesions, on hematoxylin and eosin (H&E) stained slides from accessory lung lobes (Fig. 3a,b). The high-throughput work flow presented here alleviates the heavy time burden that was previously required^34^ and incorporates the entire accessory lobe in the unbiased analysis for a more progressive and reproducible scoring system. Interestingly, Beige mice are afflicted with a significantly greater proportion of lung identified as an immune lesion area as compared to C57BL/6 mice, 4–8 weeks post infection (Fig. 3b). Indeed, accessory lobes from C57BL/6 mice have less than 10% of the area identified as lesion at 8 weeks post infection, whereas Beige mice reach 60% lesion area by 6 weeks post infection (Fig. 3b).

### In vitro growth kinetics and compound sensitivities of rough versus smooth colony morphotypes

**Figure 4:**
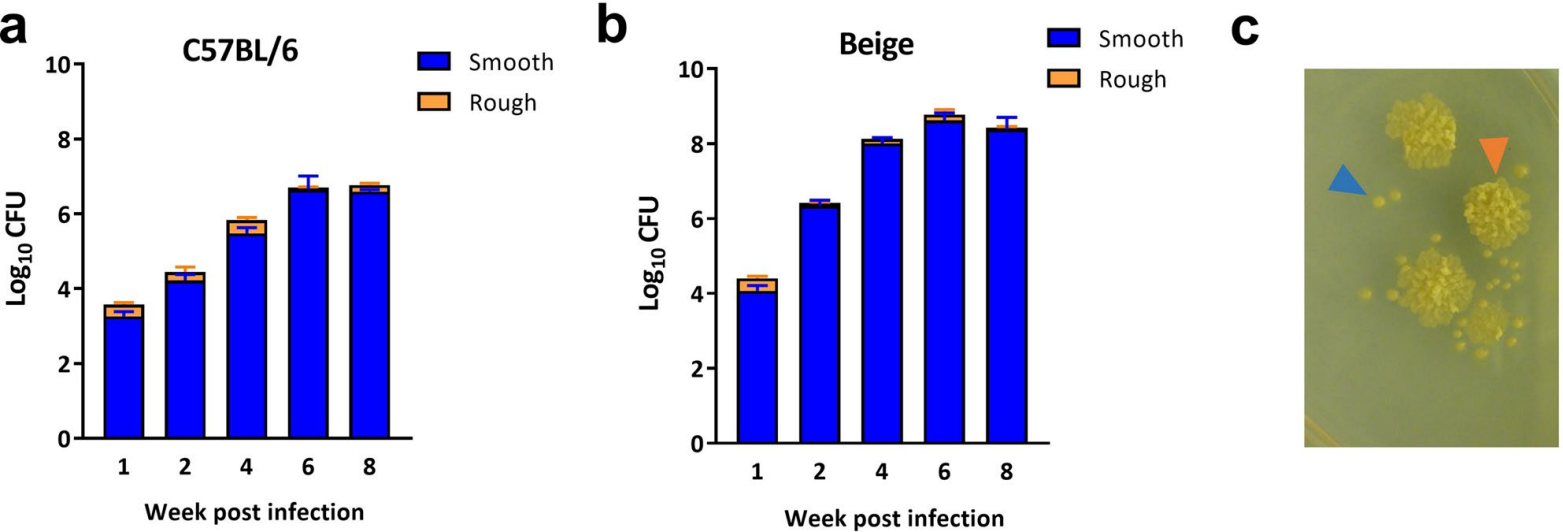
Stochastic emergence of rough colony morphotype in vivo. Proportion of smooth (blue) and rough (orange) morphotypes from lung homogenates 1–8 weeks post infection with *M. avium* 2-151 smt in (**a**) C57BL/6 and (**b**) Beige mice. (**c**) Representative image of rough (orange arrow) and smooth (blue arrow) colony phenotypes from an ex vivo lung homogenate agar plate culture.

Upon counting CFU, colony morphotypes were categorized as smooth or rough [loss or modification of glycopeptidolipid (GPL)] in appearance from ex vivo homogenate plating. While the starting infectious material was uniformly smooth transparent (smt), in both C57BL/6 and Beige mice we observed a stochastic emergence of a minor rough morphotype population (Fig. 4a–c). Interestingly, there are conflicting reports of the impact of colony morphotype on in vivo virulence. Within MAC and *M. avium* some studies suggest that rough variants (lacking GPL) induce a stronger host inflammatory response^42^ whereas other studies observed no differences in induced host cytokine production or virulence between smooth and rough phenotypes of clinical *M. avium* isolates^43,44^. This includes observations that *M. avium* rough and smooth colony morphotypes do not differentially induce proinflammatory TNF-α from human PBMCs after 20 h of in vitro infection^43^. The impact of GPL is further complicated by NTM strain variation, as studies have demonstrated divergent mouse TNF-α responses induced by two smooth variants of *M. avium*^45^*.*

Infection stock of uniform smooth morphology was sub-cultured in vitro for 5 weeks where rapid emergence of rough phenotype was not observed. Indeed, rough colonies were not noted until 3 weeks of passaging (Supplementary Table S1) in vitro whereas rough colonies were observed as early as 1 week directly ex vivo (Fig. 4).

Three rough and three smooth colonies were isolated from ex vivo lung homogenate plating and were subsequently cultured in vitro. We observed equivalent growth kinetics between smooth and rough colonies. Furthermore, when rough and smooth colonies were co-cultured in vitro the total make-up of the population retained the relative ratios of morphotypes suggesting little interference or dominance between strains in vitro (Supplementary Table S2).

There are notable differences in drug sensitivity between rough and smooth morphotypes documented for *M. abscessus* strains^26,46^, but there is little information to date for clinical *M. avium* isolates. Here we determined in vitro minimum inhibitory concentrations (MIC) by Optical Density (OD) following the addition of front-line antibiotics used to clinically treat MAC infections (Supplementary Table S3). We observed no significant difference in MIC between smooth and rough subcultures of *M. avium* 2-151 in vitro (based on a threshold of fourfold change or higher) to front line antibiotics (Supplementary Table S3)*.* Smooth and rough variants of *M. avium* 2-151 isolated ex vivo demonstrated relatively equal sensitivities to compound inhibition and direct killing.

### Vaccine candidate vetting against NTM

**Figure 5:**
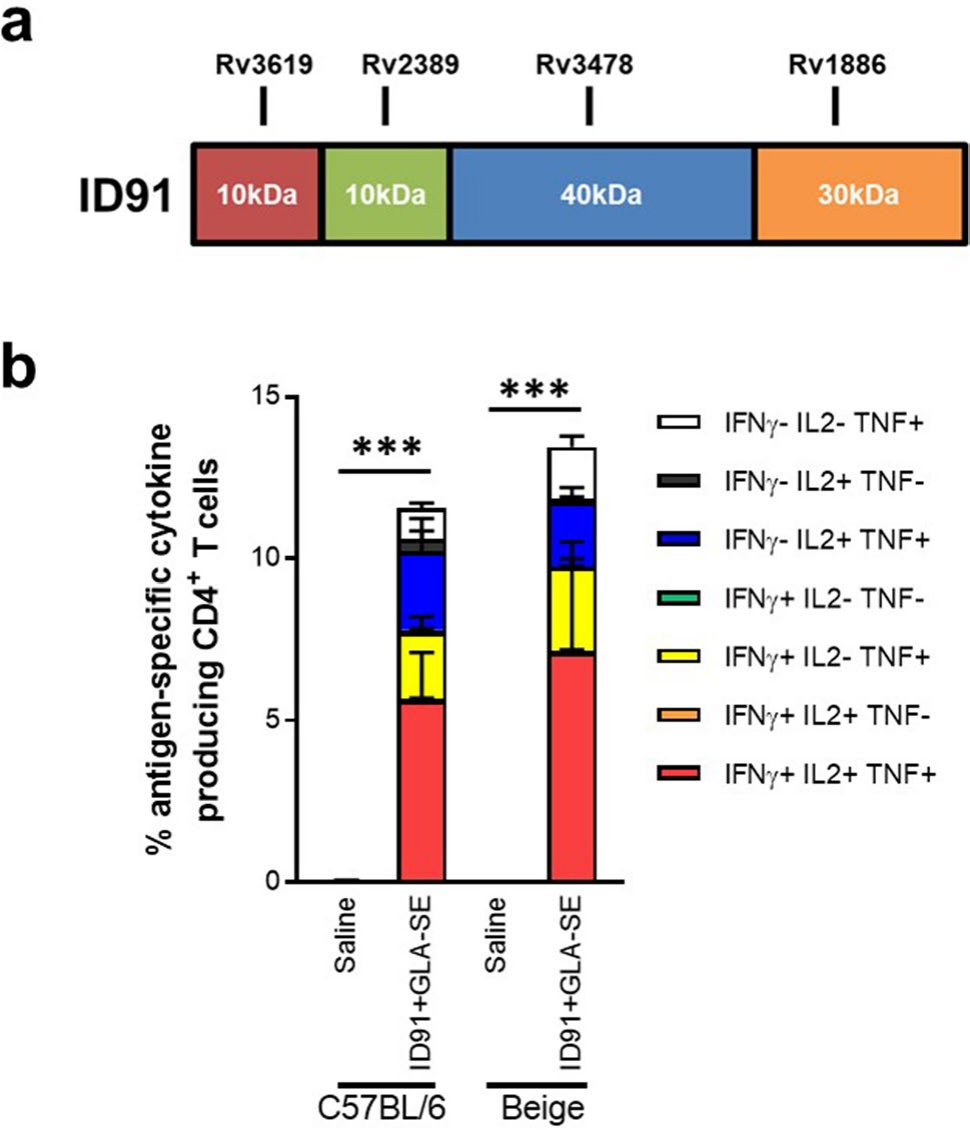
ID91 antigens are immunogenic in C57BL/6 and Beige mice. (**a**) Mtb antigens that make up the subunit fusion antigen ID91. (**b**) Percent of polyfunctional CD4^+^ T cells restimulated with ID91 antigens ex vivo from C57BL/6 or Beige mice immunized with saline or ID91 + GLA-SE, two times three weeks apart. Data represent average ± SD of n = 4/group, representative of two independent experiments.

Preclinical Mtb vaccine candidate, ID91, is a fusion of four Mtb proteins: Rv3619 (esxV; ESAT6-like protein), Rv2389 (RpfD), Rv3478 (PPE60) and Rv1886 (Ag85B) (Fig. 5a). These antigens were selected as candidates for Mtb vaccines based on their lack of human sequence homology and their ability to induce an ex vivo IFN-γ response in PPD + human peripheral blood mononuclear cells (PBMC), suggesting they are immunogenic in humans^47,48^. Despite being derived from Mtb, these antigens share significant sequence homology amongst different mycobacterial species, including *M. avium* (Table 1). Indeed, when we executed a theoretical epitope prediction, using the IEDB database, we observed significant overlap of predicted MCHII binding peptides in the mouse H2-iAB allelic background (Supplemental Table S4–S7) as well as predicted immunogenic CD4^+^ T cell epitopes across human HLAs (Supplemental Table S8). Not surprisingly, the greatest overlap in these predicted epitopes were between Rv3619 and Rv1886 antigen homologs given they have the highest sequence identity between Mtb and *M. avium*.

**Table 1:** Mycobacterial consensus of ID91 antigens.

Comparison to *M. avium* homolog	Mtb antigen			
	Rv3619	Rv2389	Rv3478	Rv1886
Percent identity	88	49	47	85
Percent positive	91	62	61	92
e-value	4e-62	1e-36	1e-57	0.0

Values reported based on NCBI BLASTp of protein sequences from Mtb and *M. avium* antigen alignments.

Therefore we leveraged this ID91-based vaccine as a proof-of-concept subunit candidate against *M. avium* 2-151 smt challenge in the preclinical model. Interestingly, T cell responses directed to Ag85B, an antigen in the vaccine candidate, have demonstrated some protective efficacy against *M. avium* in a P25 transgenic mouse model (the majority of TCR specific for Ag85B) akin to that observed in C57BL/6 wild type mice^49^. Moreover, we verified that ID91 antigens formulated in GLA-SE were immunogenic in both Beige and C57BL/6 mice. Vaccine candidate ID91 + GLA-SE was given twice intramuscularly two weeks apart and splenocytes were isolated one week after the boost immunization. Robust CD4^+^ T helper 1 responses (T_H_1), including production of IFN-γ, IL-2 and TNF cytokines, were elicited from ex vivo ID91 restimulated splenocytes from ID91 + GLA-SE immunized mice but not from mice that received saline only (Fig. 5b, Supplemental Figure S3).

**Figure 6:**
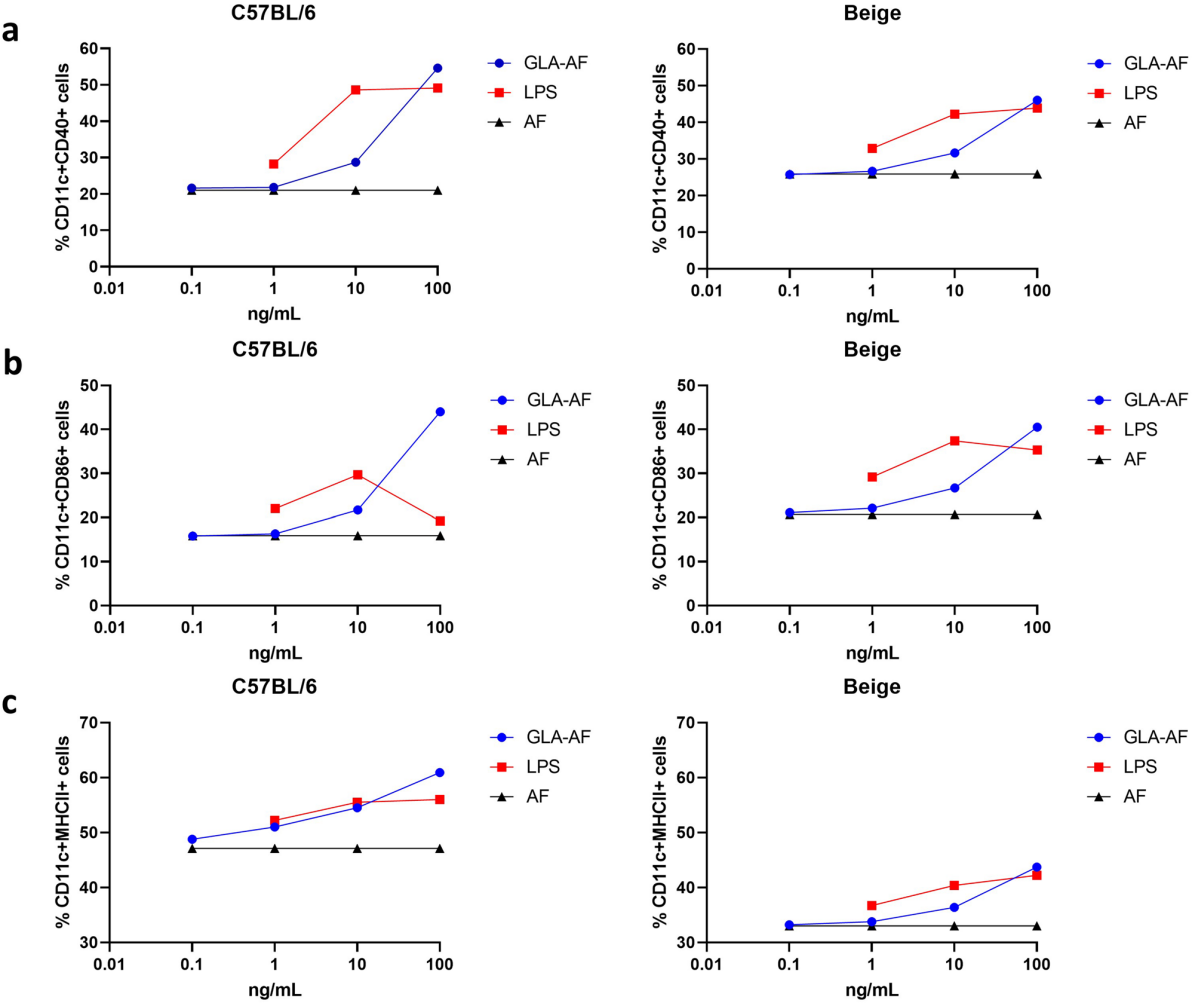
Activation of BMDC with GLA innate immune modulator. BMDC from C57BL/6 (left) and Beige (right) were stimulated with increasing concentrations (0.1–100 ng/mL) of GLA-AF (blue circles) and LPS (red squares) or equivalent volumes of AF (black triangles), and after 48 h surface stained for activation markers. CD11c positive BMDC cells were evaluated for the percentage of cells co-expressing (**a**) CD40, (**b**) CD86 and (**c**) MHCII.

We next evaluated whether each mouse strain was equally sensitive and receptive to innate immune stimulation by the synthetic TLR4 agonist, GLA, by evaluating surface expression of activation and costimulatory markers on mouse bone marrow-derived dendritic cells (BMDC). As we have observed previously^50^, GLA induced expression of CD40 and CD86 activation markers on CD11c^+^ cells in a dose-dependent manner (Fig. 6a,b). The percentage of CD40 and CD86 expression induced by LPS and GLA-aqueous formulation-(AF) was similar between C57BL/6 and Beige CD11c^+^ BMDC (Fig. 6a,b). Although we detected no global deficiency in BMDC GLA-induced activation between mouse strains we did observe dramatically lower expression of MHCII (Fig. 6c) in Beige mice across the concentrations of innate immune modulators tested compared to similar doses evaluated in C57BL/6 mice.

### ID91 + GLA-SE and BCG immunizations induce T_H_1 and humoral responses in both WT and immunocompromised mouse models

Bacillus Calmette–Guérin (BCG), the only licensed Mtb vaccine, has demonstrated cross-reactive immune responses against *M. avium* in both human PBMC and mouse lymphocytes^51^, and is protective against challenge with *M. avium* in mice^52–54^. Furthermore, retrospective epidemiological evidence from Finland suggests that cessation of universal BCG administration in infants across the nation has coincided with a significant increase of NTM infection and disease in children^55^. Route of administration of BCG is an active and promising area of research, including the exciting publication by P. A. Darrah and colleagues whereby intravenous (i.v.) and intradermal (i.d.) BCG immunizations in nonhuman primates were evaluated for efficacy against Mtb challenge^56^. In our mouse preclinical model, as well as those of our colleagues, BCG given i.d. has proven to be a moderately effective vaccine against Mtb^34,57–60^. Therefore, we utilized an i.d. administration of BCG as a positive control for our vaccine efficacy and immunogenicity evaluations.

**Figure 7:**
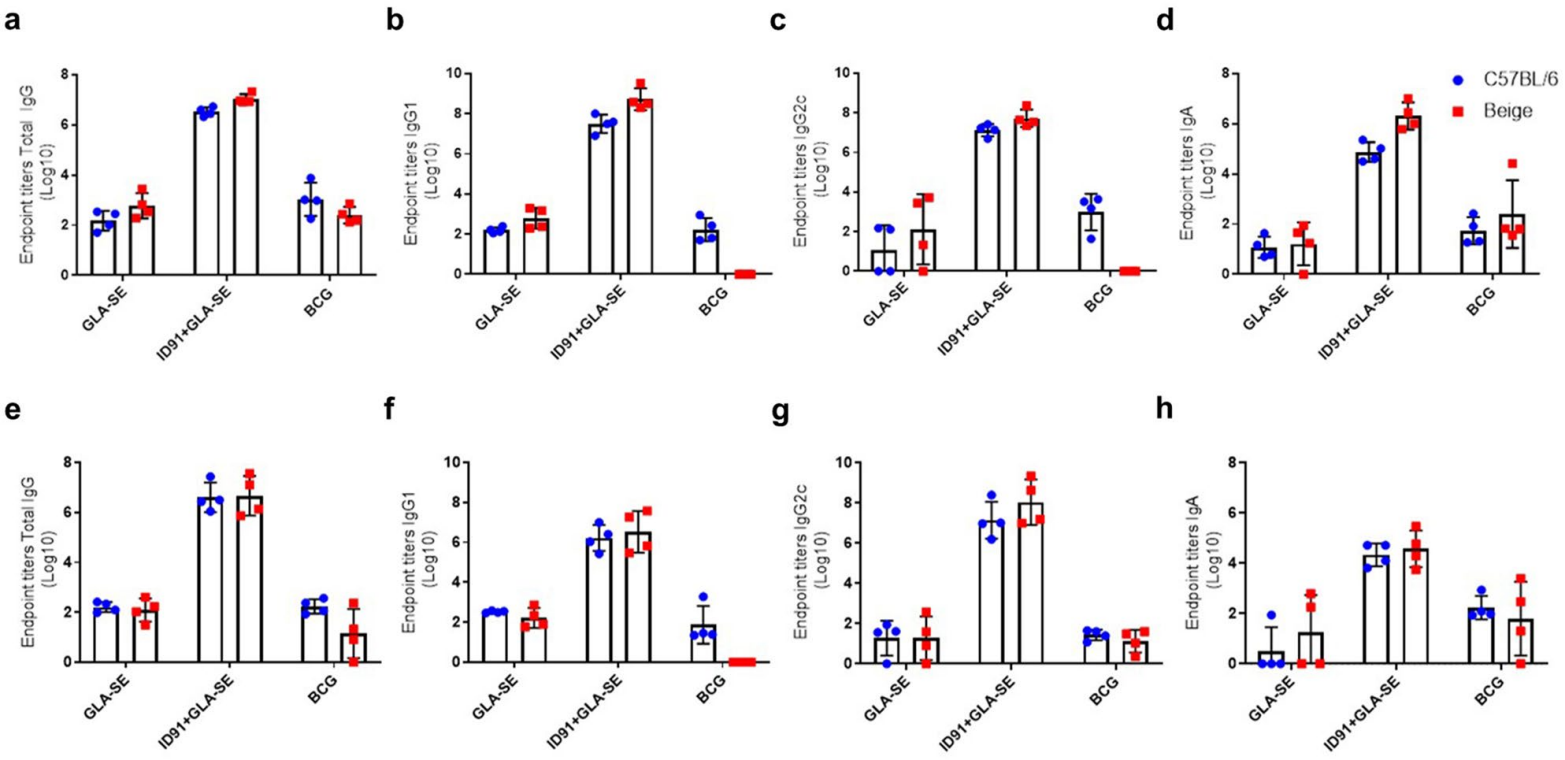
Humoral responses in C57BL/6 and Beige mice six weeks post-infection with *M. avium* 2-151 smt. Serum from prophylactically immunized C57BL/6 (blue circles) and Beige (red squares) mice with GLA-SE, ID91 + GLA-SE, or BCG was evaluated for ID91 antigen specific (**a**) total IgG, (**b**) IgG2c, (**c**) IgG1 and (**d**) IgA endpoint titers as well as Ag85B-specific (**e**) total IgG, (**f**) IgG2c, (**g**) IgG1 and (**h**) IgA endpoint titers. Bars represent average endpoint titers ± SD, individual shapes represent individuals, n = 4/group.

Six weeks post infection, vaccine immunogenicity (humoral and cellular) was evaluated in cohorts of mice that were immunized with adjuvant alone (GLA-SE), ID91 + GLA-SE or BCG and challenged with a LDA of *M. avium* 2-151 smt. C57BL/6 and Beige mice immunized with ID91 + GLA-SE demonstrated robust ID91 antigen-specific (Fig. 7a–d) and Ag85B-specific (Fig. 7e–h) total IgG, IgG2c, IgG1, and IgA antibody responses, whereas GLA-SE or BCG immunized mice demonstrated much weaker ID91 and Ag85B antigen-specific humoral responses. Interestingly, C57BL/6 and Beige mice seemed to induce a similar degree of each antibody subtype within each immunization cohort.

**Figure 8:**
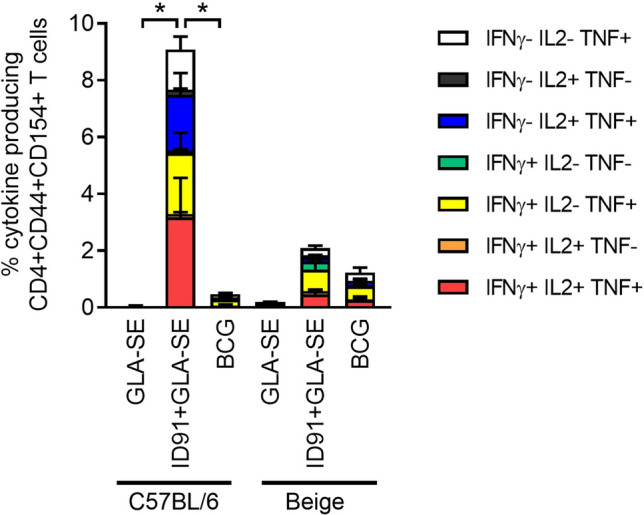
Greater magnitude and composition of antigen-specific polyfunctional CD4^+^ T cells with ID91 + GLA-SE vaccination. Pulmonary T_H_1 cytokine producing CD4 + CD44 + CD154 + T cells 6 weeks post *M. avium* 2-151 smt challenge from immunized C57BL/6 or Beige mouse cohorts restimulated ex vivo with ID91. *p < 0.05, one-way ANOVA with Tukey's multiple comparison test.

Lymphocytes were isolated from immunized cohorts six weeks post challenge from individual lung homogenates and restimulated ex vivo with ID91 antigens (Fig. 8, Supplemental Figure S3). These cells were then stained and evaluated by flow cytometry for CD4^+^ T_H_1 responses. The magnitude and composition of antigen specific polyfunctional (expressing IFN-γ, IL-2, TNF or a combination of these cytokines) CD4^+^ T_H_1 T cells was significantly higher in ID91 + GLA-SE immunized C57BL/6 mice than BCG or GLA-SE immunized C57BL/6 mice (Fig. 8). Interestingly this pattern was much less robust and not significant for Beige mouse cohorts.

### ID91 + GLA-SE and BCG prophylactically protect WT and immunocompromised mice from *M. avium* 2-151 smt bacterial burden

**Figure 9:**
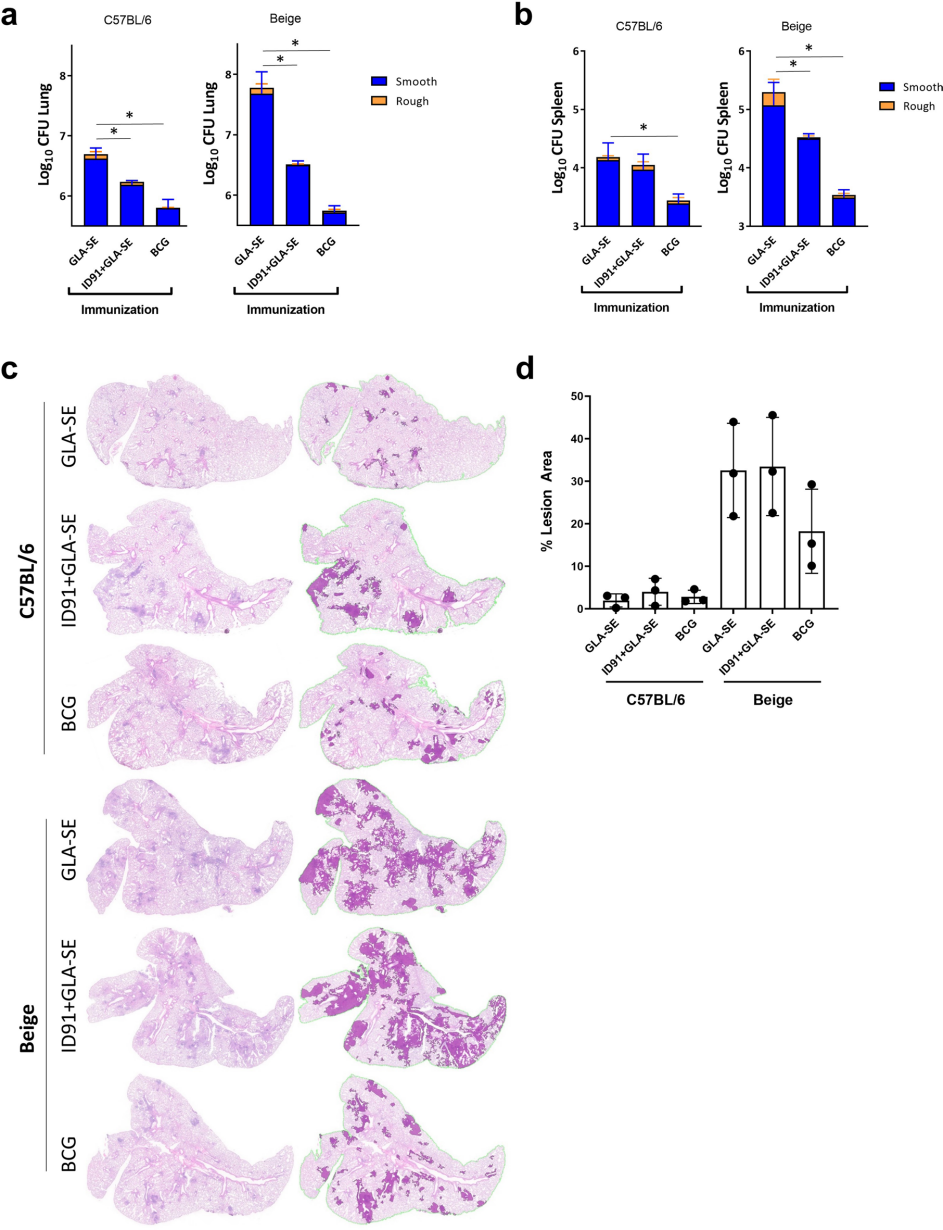
ID91 + GLA-SE and BCG prophylactically protect against pulmonary *M. avium* 2-151 smt infection. Cohorts of C57BL/6 and Beige mice were prophylactically immunized i.m. with GLA-SE, or ID91 + GLA-SE or i.d. with BCG and subsequently challenged with *M. avium* 2-151 smt. Bacterial burden in (**a**) lung and (**b**) spleen 6 weeks (42 days) post infection with *M. avium* 2-151 smt in C57BL/6 and Beige mice. Mean CFU ±SD for n = 7/group. *p < 0.05, significant by one-way ANOVA of total CFU. (**c**) Representative accessory lung lobes from C57BL/6 (top) and Beige (bottom), before (left column) and after (right column) algorithmic scoring of H&E staining for pulmonary lesions (dark purple). (**d**) Mean ± SD percent lesion area, n = 3/group per time point. n.s. by ANOVA with Sidak’s multiple comparison test between treatments within strains.

Vaccination with ID91 + GLA-SE or BCG induced significant prophylactic protection as observed by a reduction of bacterial burden (consisting of both rough and smooth morphotypes, albeit a predominately higher proportion of smooth colonies), in the lungs and spleens of C57BL/6 and Beige mice (Fig. 9a,b). Excitingly, BCG and ID91 + GLA-SE reduced lung bacterial burden in the lungs of Beige mice by 1.76 and 0.77 Log_10_ CFU, respectively, compared to adjuvant alone (Fig. 9a). Interestingly, there was no significant difference in pulmonary lesion area at 6-weeks post infection for any treatment group of C57BL/6 mice, with relatively low (< 10%) lesion scores (Fig. 9c,d). Conversely, Beige mice demonstrated significantly higher lesion area compared to C57BL/6 across all treatment arms. While no treatment was significant, prophylactic BCG immunization did trend with lower immunopathology in Beige mice (Fig. 9c,d).

A pattern of vaccine-induced protection was independently obtained from parallel studies performed by our collaborators, demonstrating significant ID91 + GLA-SE vaccine-induced protection against *M. avium* 2-151 smt in the C57BL/6 model 6 weeks post challenge (Supplementary Figure S4). These data suggest vaccination, even in an immunocompromised host setting, may be a viable approach for protection from NTM.

## Conclusions

In this work, we observed a sustained infection in wild type (C57BL/6) and immunocompromised (Beige) mouse models using a virulent clinical isolate of *M. avium*. These representative mouse models will be useful for developing candidate vaccines targeting NTM in immunocompromised or immune competent individuals. Beige mice sustain a higher bacterial burden over time than C57BL/6 mice and demonstrate a differential pulmonary immune cell influx post challenge. C57BL/6 mice exhibit earlier and more robust monocyte and neutrophil pulmonary influx 24 h to 2 weeks post challenge, as well as an increase in lymphocytes 6 and 8 weeks post challenge compared to Beige mice, and considerably less immunopathology over time which may be directly related to their divergent ability to control infection early and late. These data represent an expansion on previous reports^40,41^, including the aerosol route of challenge and the specific concurrent quantification of lymphocytes, mononuclear phagocytes, NK cells, and neutrophils in the lungs of infected mice over time. While Beige mice do not represent a model that is defective in proinflammatory signaling axes, which can predispose humans to NTM infection, this mouse strain provides a unique opportunity as a model, where these mice can (1) sustain infection out to at least 6 weeks post challenge with NTM in spite of their slight immunodeficiencies, and can (2) still be further immunologically manipulated (e.g. cell depletion) to extrapolate vaccine-induced mechanisms of control/resistance. Interestingly, we observed a stochastic rough-colony phenotype emergence in vivo as early as 1 week following infection with *M. avium* 2-151smt, whereas a switch to rough morphology was less prominent in vitro suggesting that in vivo pressure(s) may be critical for this loss/modification to GPL. There was no evidence of interference between morphotypes in vitro or preferential growth kinetics. Furthermore, we did not identify differences in sensitivity to front line antibiotics in vitro between rough and smooth *M. avium* 2-151 morphologies.

Our proof of concept vaccine candidate ID91 + GLA-SE was readily immunogenic in both mouse models, including enhanced ID91-specific humoral and CD4^+^ T_H_1 cellular responses. This is an important finding given that IFN-γ activates neutrophils and macrophages to phagocytose and/or kill intracellular pathogens, including mycobacteria such as NTM^61–64^. Furthermore, the positive feedback loop between IFN-γ and IL-12 is critical for the control of mycobacteria^65,66^. Cellular CD4^+^ T_H_1 T cell responses are likely a critical component for mycobacterial infection susceptibility as persons living with HIV and waning CD4^+^ T cell counts (< 50 cells/µl) can present with disseminated extrapulmonary Mtb or NTM infections^67,68^. TNF, another prominent T_H_1 cytokine, has been clinically shown to help control NTM infections, which if inhibited, as with other mycobacterial infections, can result in increased risk of infection^8,69–71^. In cases where a genetic deficiency in the IL-12/IFN-γ signaling axis leads to enhanced risk of NTM infection, other T_H_1 cytokines like TNF may be able to fill this void and reduce the overall susceptibility. The complete mechanisms of protection against NTM infection are not yet known^17^. Therefore, we hypothesized that a T_H_1 skewing vaccine candidate may afford partial protection in WT and immunocompromized preclinical models.

Indeed, both ID91 + GLA-SE and BCG demonstrate prophylactic protection against *M. avium* 2-151 smt aerosol challenge. Importantly, BCG immunization resulted in greater protection in both Beige and C57BL/6 mice compared to the proof of concept ID91 + GLA-SE vaccine, emphasizing the need for more work in specific antigen design and optimization for future candidates. In these experiments, pulmonary lesion scores did not align with overall bacterial burden, suggesting the mechanism of protection may well involve immune cell kinetics and be less directly related to maintaining pulmonary architecture. Although BCG was leveraged in this study as a positive control, historically live-attenuated vaccines have been contraindicated for immune compromised individuals, the same population disproportionately affected by NTM disease. BCG is not recommended for individuals living with HIV/AIDS as BCG-induced disseminated disease has been reported^72,73^. Furthermore, while BCG vaccination before NTM exposure induces some cross-reactive immunity^51^, BCG vaccination in the preclinical model after infection or exposure to NTM does not seem to reduce NTM infection or disease^74^. The complex interplay of historical mycobacterial exposures, both repeatedly to environmental NTM and BCG vaccination, is an important consideration for early vaccine design and efficacy testing. We believe that through rational vaccine platform selection and design a vaccine against NTM and specifically *M. avium*, can be developed as a safe strategy against this opportunistic infection.

Although outside the scope of this proof-of-concept study, it is not clear whether BCG and a subunit vaccine could provide synergistic protection against NTM. Other vaccine platform approaches, including DNA-based vaccines encoding mycobacterial antigen^52,75^, have not yet demonstrated synergistic protection over that of BCG alone in the preclinical model, despite inducing significant antigen-specific IFN-γ^+^ CD4^+^ T_H_1 responses^52,53^. *M. avium* culture filtrate protein (CFP) has also been explored as a heterologous mixed protein vaccine candidate, akin to Mtb studies^76^, and when formulated with DDA adjuvant was able to afford 1.0 and 0.5 Log_10_ CFU protection in the liver and spleen, respectively, in an intravenous *M. avium* BALB/c challenge model^66^. The work detailed here further demonstrates that subunit + adjuvant vaccine platforms are a viable approach for novel or complimentary interventions against *M. avium*.

As a rich collection of species with expanding prevalence and global health burden, research focus on NTM should be prioritized. Vaccine strategies that (1) develop NTM-specific antigen candidates that are immunogenic in humans and (2) devise focused regimens with respect to different disease indications (e.g. Cystic Fibrosis (CF) or anti-TNF-therapy, scenarios with defects in mechanical or physical disruptions to the pulmonary space as well as those with disrupted proinflammatory signaling), have the capacity to significantly impact the burden of NTM disease. Moreover, the successful development of a vaccine against NTM species may help inform vaccines against other mycobacteria, such as Mtb.

## Materials and methods

### Preclinical animal model

Female C57BL/6 mice and Beige mice 4–6 weeks of age were purchased from Charles River Laboratory and Jackson Laboratory (Sacramento, CA, USA), respectively. All methods were carried out in accordance with relevant guidelines and regulations with respect to animal welfare. Mice were housed at the Infectious Disease Research Institute (IDRI) biosafety level 3 animal facility under pathogen-free conditions and were handled in accordance with experimental protocols that were approved by IDRIs Institutional Animal Care and Use Committee (IACUC). Mice were infected either by the intranasal (i.n.) route or aerosol challenge with 10^4^ CFU of *Mycobacterium avium* Chester or *Mycobacterium avium* 2-151 smt^77^ (obtained from Dr. Diane Ordway of CSU) using a University of Wisconsin-Madison aerosol whole body exposure chamber with a Collison nebulizer. Twenty-four hours post challenge the lungs of three mice were homogenized and plated on Middlebrook 7H10 agar (Molecular Toxicology, Boone, North Carolina, USA) to confirm delivery quantity of 10^4^ CFU per mouse. Other Female C57BL/6 mice 4–6 weeks of age were housed at Colorado State University (CSU) biosafety level 3 animal facility in accordance with the CSU IACUC. These mice were used in repeat vaccination experiments as described below or to evaluate other clinical MAC isolates for bacterial burden over time. Some of these animals were challenged via aerosol with *M. avium* 101^78^ (obtained from ATCC), *M. avium* 2285 S or *M. avium* 2285 R^38^ (obtained from Dr. Kenneth N. Oliver of the NIH Pulmonary Clinical Medicine Section) using a Glas-Col whole body exposure chamber.

### Bacterial burden/CFU

Bacterial counts were enumerated from 4 to 7 mice per group, 24 h to 8 weeks post infection. With methodology we have previously reported^34^, mice were euthanized with CO_2,_ and lung and spleen tissue from infected animals was isolated and homogenized in 5 mL of either RPMI + FBS (lung) or PBS + Tween-80 (Sigma-Aldrich, St. Louis, Missouri, USA) CFU buffer (spleen) using an Omni tissue homogenizer (Omni International, Kennesaw, GA, USA) or Precellys Tissue Homogenizer (Bertin Corp. Rockville, MD, USA). Serial dilutions of homogenate were made in CFU buffer and aliquots were plated on Middlebrook 7H10 agar plates and subsequently incubated at 37 °C and 5% CO_2_ for 2–3 weeks before colonies were counted. Bacterial burden, as CFU/mL, was calculated per organ and is presented here as Log_10_ values. Colony morphotype (rough versus smooth appearance) was also denoted at counting. Reduction in the bacterial burden was calculated as the difference in mean Log10 values between groups assessed.

### Histopathology and image analysis

Lung accessory lobes were collected at different time points after *M. avium* 2-151 smt challenge and were immediately perfused and subsequently stored in 10% normal buffered formalin. Fixed lungs were embedded in paraffin, cut and slides were generated with hematoxylin and eosin (H&E) staining at the Benaroya Research Institute Histology Core facility (Seattle, WA, USA), with methodology previously reported^34^. Stained slides were blinded and analyzed by a veterinary pathologist (Dr. Brendan Podell, Colorado State University). H&E stained sections were scanned at 20X magnification using an Olympus VS120 microscope, Hamamatsu ORCA-R2 camera, and Olympus VS-ASW 2.9 software. Visiopharm software was used for image analysis. For each tissue section, a region of interest (ROI) was generated at a low magnification with a custom tissue detecting algorithm using decision forest training and classification to differentiate tissue versus background based on color and area. Lesions were identified within tissue ROI’s at a high magnification with an additional custom-made algorithm using decision forest training and classification based on staining intensity, color normalization and deconvolution, area, and morphological features. Percent lesion calculations were integrated into the same algorithm and calculated from tissue area and lesion area as designated by the ROI and lesions detected. Lesion identification and quantification were then reviewed and edited by a pathologist as needed^79^.

### In vitro growth kinetics

*M. avium* 2-151 smooth and rough colony variants were isolated from ex vivo lung homogenate plating. These isolates were grown in Middlebrook 7H9 medium supplemented with 10% v/v OADC (oleic acid, albumin, dextrose, catalase) and 0.05% w/v Tween 80 (7H9-Tw-OADC) under aerobic conditions.

### In vitro growth of *M. avium* smooth and rough variants

*M. avium* 2-151 smt was inoculated at an OD_590_ of 0.05 into a stirring culture of 7H9-Tw-OADC. Cultures were diluted 1:10 weekly in fresh 7H9-Tw-OADC and serial dilutions were plated onto 7H10-OADC agar plates to look for emergence of a rough variant. For competition experiments, *M. avium* 2-151 smooth and rough variants were inoculated at various ratios (based on OD_590_) into 7H9-Tw-OADC. Cultures were diluted 1:10 weekly in fresh 7H9-Tw-OADC and serial dilutions were plated onto 7H10-OADC agar plates. Smooth and rough colonies were counted and converted to a percentage of the overall colony count.

### Determination of minimum inhibitory concentration (MIC)

MICs of selected anti-bacterial compounds were assessed with minor modifications as previously reported^80^. Briefly, MICs were determined against *M. avium* 2-151 smooth or *M. avium* 2-151 rough morphotypes grown in 7H9-Tw-OADC under aerobic conditions. Compounds dissolved in dimethylsulfoxide (DMSO) were added to 96 well plates using automated liquid handlers to a final DMSO concentration of 2%. Bacteria was added to a starting OD_590_ of 0.02 and growth was measured by OD_590_ after 5 days of growth at 37 °C. MIC is defined as the concentration of compound required to inhibit growth of *M. avium* by 90% and was determined from the Levenberg–Marquardt least squares plot^81^.

### Determination of compound killing kinetics

*M. avium* smt or *M. avium* rough variants were inoculated at ~ 1 × 10^5^ colony-forming units (CFU)/mL into 7H9-Tw OADC containing compound or DMSO alone as a control (final DMSO concentration of 2% in all samples). Standing cultures were incubated for 7 days at 37 °C, and serial dilutions were plated for CFU determination.

### ID91 antigen comparison

Mtb antigens that comprise the fusion ID91 were aligned to homologs from *M. avium* using NCBI BLASTp and the compositional matrix adjust method to generate e-values, percent identity and percent positive comparisons between species of mycobacteria. The MHCII binding predictions made for each antigen were made on 12/15/2020 using the IEDB analysis resource Consensus tool^82,83^ (tool.iedb.org), using mouse H2-iAB allele selection with the consensus (smm/nn) method and default 15-mer length. Low adjusted rank is considered good potential binders, and a maximum threshold of 50 was used. We also leveraged the IEDB CD4^+^ T cell Immunogenicity prediction tool^84^ using combined prediction methods and a maximum combined score threshold of 50 across human HLAs.

### Vaccines and adjuvants

Cohorts of mice were immunized intramuscularly (i.m.) three times three weeks apart, with the final immunization occurring 4 weeks before challenge. Mice received either saline alone, or vaccinations containing 0.5 µg/dose of ID91 recombinant fusion protein combined with 5.0 µg/dose TLR4 stimulant glucopyranosyl lipid adjuvant (GLA), formulated in a squalene emulsion (SE), as previously published^34,50,85^. A separate cohort of mice were immunized once intradermally (i.d.) with 10^4^ CFU of bacillus Calmette–Guérin (BCG) (Sanofi Pasteur, Theracys) 10 weeks before challenge; mice received 100 µl total, 50 µl on either side of the base of the tail.

### Bone marrow-derived DC

BMDC were culture-induced in vitro using sterile aspirated bone marrow from the femurs of Beige and C57BL/6 mice. BMDC were prepared as previously published^50^. Briefly, bone marrow cell suspensions were prepared, washed and suspended at a concentration of 10^6^ cells/mL in complete medium (RPMI 1640, 10% fetal bovine serum, 1% L-glutamine, 1% penicillin/streptomycin) supplemented with 20 ng/mL recombinant mouse GM-CSF and 20 ng/mL IL-4 (PeproTech, Rocky Hill, NJ). On day 3, DC were supplemented with complete media and 20 ng/mL GM-CSF. On day 6, non-adherent cells were plated at 2 × 10^5^ cells/well in fresh complete RPMI medium, and stimulated for 48 h at 37 °C with 0.1–100 ng/mL of GLA, LPS or aqueous formulation (AF) agonists as indicated in the figure legends. After 48 h cells were surface stained with the following antibodies: CD11c (Bv650, clone N418, Biolegend), CD86 (APC, clone GL-1, Biolegend), CD40 (PE/Cy7, clone 3/23, Biolegend), and MHCII I-A/I-E (eF450, clone M5/114.15.2, Invitrogen), and analyzed by flow cytometry as described below.

### Antibody ELISAs

Serum was collected from mice immediately after euthanasia, six weeks post challenge. Serum was diluted 1:100 (or 1:10 for IgA) and added to plates coated with either 2 µg/mL of ID91 antigen or 2 µg/mL of Ag85B. After overnight incubation, plates were exposed to HRP-conjugated total IgG, IgG1, IgG2c, or IgA (Southern Biotech). Plates were subsequently developed with SureBlue tetramethylbenzidine substrate (KPL/Seracare) and the reaction stopped with 1 N H2SO4. Plates were read at 450 nm with 570 nm background subtraction.

### Flow cytometry

Intracellular flow cytometry was performed on lung homogenates 24 h to 8 weeks post *M. avium* infection with methodology as previously reported^34^. Specifically, samples were incubated in red blood cell lysis buffer (eBioscience/Thermo Fischer Scientific, Waltham, Massachusetts, USA), washed and resuspended in RPMI 1640 (Life Technologies, Carlsbad, California, USA) + 10% fetal bovine serum (FBS) (BioWhittaker, Inc./Lonza Radnor, Pennsylvania, USA), and subsequently aliquotted in 96-well round-bottom plates. Cells were then stimulated with media alone, 10 µg/mL of recombinant ID91, or 1 µg/mL phorbol myristate acetate (PMA) (Calbiochem) + 1 µg/mL ionomycin (Sigma-Aldrich, St. Louis, Missouri, USA) and incubated at 37 °C. After two hours of incubation, 1 µg/µL of GolgiPlug (BD Biosciences) was added and samples were incubated at 37 °C for an additional 8 h. Samples then remained at 4 °C until staining. Samples were first surface stained with fluorochrome-conjugated antibodies against mouse CD4 (Bv650, clone RM4-5, BioLegend, San Diego, California, USA), CD8 (Bv510, clone 53-6.7, BioLegend), CD44 (APC-eF780, clone IM7, Invitrogen) and 1 µg/mL of Fc receptor block anti-CD16/CD32 (clone 93, eBioscience) in PBS with 1% bovine serum albumin (BSA) for 10–15 min at room temperature (RT). Cells were then washed and fixed using BD Biosciences Fix/Perm reagent for 20 min at RT. Subsequently, samples were washed with BD Perm/Wash followed by intracellular staining in Perm/Wash reagent with anti-mouse IFN-γ (PE-Cy7, clone XMG1.2, Invitrogen), IL-2 (APC, clone JES6-5H4, eBioscience), TNF-α (eF450, clone MP6-XT22, eBioscience), and CD154 (PerCP-710, clone MR7, eBioscience) for 15 min at RT. Early influx of mononuclear phagocytes, neutrophils and lymphocytes were assessed in lung homogenates 1–8 weeks post infection. Cells were surface stained with flourochrome-conjugated antibodies against mouse Ly6G (FITC, clone 1A8, Biolegend), Ly6C (PerCP-Cy5.5, clone HK1.4, eBioscience), MHCII I-A/I-E (eF450, clone M5/114.15.2, Invitrogen), CD11c (Bv510, clone N418, Biolegend), CD3 (Bv650, clone 17A2, Biolegend), CD19 (APC, clone 6D5, Biolegend), CD11b (Alexa700, clone M1/70, eBioscience), NK1.1 (PE, clone PK136, eBioscience), CD64 (PE-Cy7, clone X54-5/7.1, Biolegend) and 1 µg/mL of Fc receptor block anti-CD16/CD32 (clone 93, eBioscience) in PBS with 1% bovine serum albumin (BSA) for 10–15 min at room temperature (RT). All antibodies were used at 10 µL/mL. Before removing samples from the BSL3, samples were incubated in 4% paraformaldehyde for 20 min. After wash and resuspension in PBS + 1% BSA, samples were acquired on a BD Bioscience LSRFortessa flow cytometer (BD Bioscience). Gating strategies were based on our previous publications^34^ and work by Yen-Rei A. Yu and colleagues^86^.

### Statistics

Intracellular cytokines induced after T cell restimulation, and bacterial burden over time were compared between groups using two-way ANOVA with Sidak’s multiple comparison test. Bacterial burden at a single time point was assessed using one-way ANOVA with Sidak’s multiple comparison test compared to groups immunized with adjuvant alone. Flow cytometry data was assessed using FlowJo v10 (BD) and SPICE (NIH) using the Wilcoxon signed rank test. Statistical analyses, aside from flow cytometry, were performed using GraphPad Prism 7 software. p values < 0.05 were considered significant and labeled accordingly in the figures, with methodology our group has previously reported^34^.

## Supplementary Information

Supplementary information.

## Data Availability

The datasets generated and/or analyzed during the study presented here are available from the corresponding authors on reasonable request.
