# Supplementary material for: Subunit vaccine protects against a clinical isolate of Mycobacterium avium in wild type and immunocompromised mouse models
Source: Sci Rep. 2021 Apr 27;11:9040. doi: 10.1038/s41598-021-88291-8 (PMC8079704; doi:10.1038/s41598-021-88291-8)
Supplement: Supplementary file 1 — Supplementary information. [file 41598_2021_88291_MOESM1_ESM.docx]

**Subunit vaccine protects against a clinical isolate of *Mycobacterium avium* in wild type and immunocompromised mouse models**

Sasha E. Larsen^1^, Valerie A. Reese^1^, Tiffany Pecor^1^, Bryan Berube^1^, Sarah K. Cooper^2^, Guy Brewer^3^, Diane Ordway^2^, Marcela Henao-Tamayo^2^, Brendan K. Podell^2^, Susan L. Baldwin^1*^, Rhea N. Coler^1*^.

^1^Seattle Children’s Research Institute, Center for Global Infectious Disease Research, Seattle, WA

^2^Department of Microbiology, Immunology and Pathology, Colorado State University, Fort Collins, CO

^3^Alternative Behavior Strategies Inc., Salt Lake City, Utah

*Correspondence to rhea.coler@seattlechildrens.org and/or susan.baldwin@seattlechildrens.org

**Supplementary Figure 1**

**
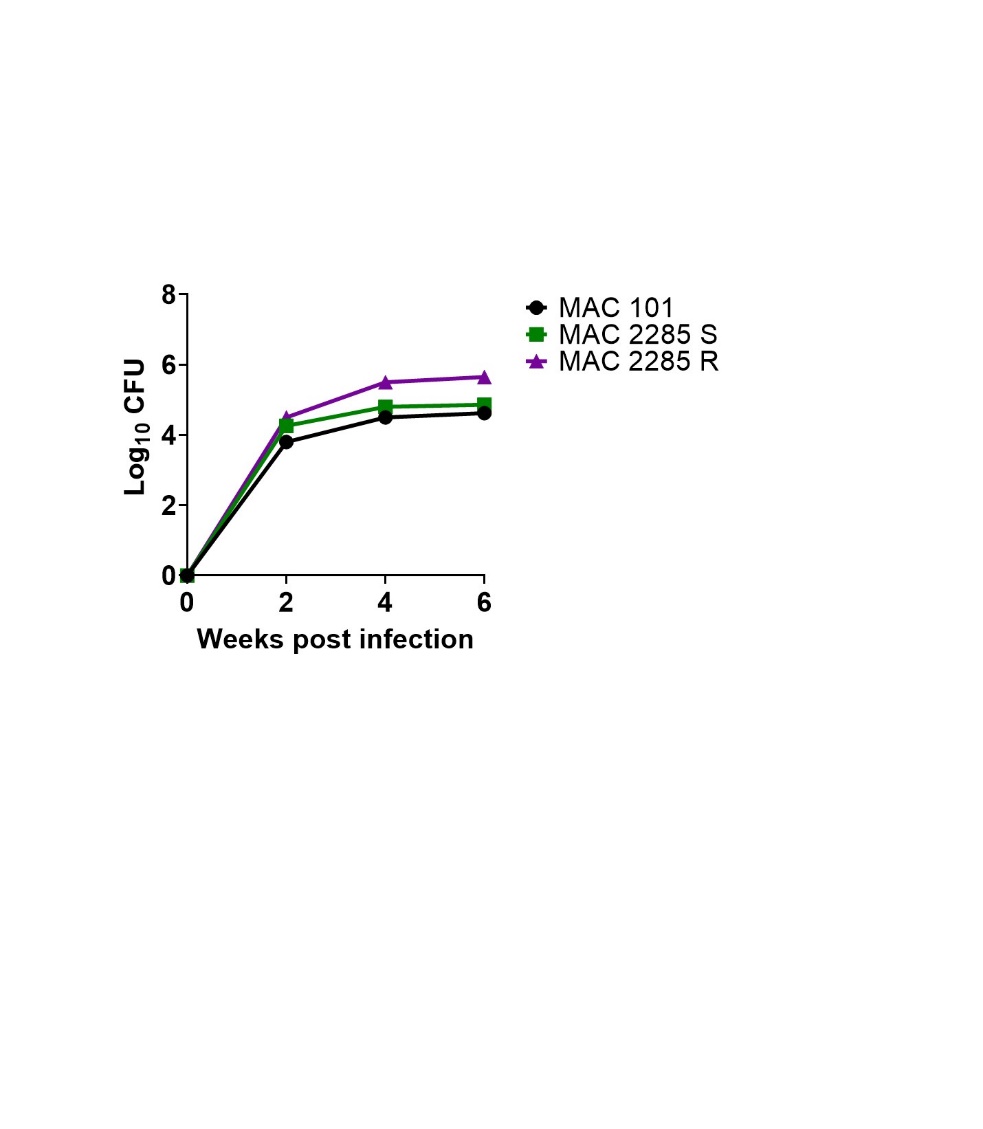
**

**Supplementary Figure 2**


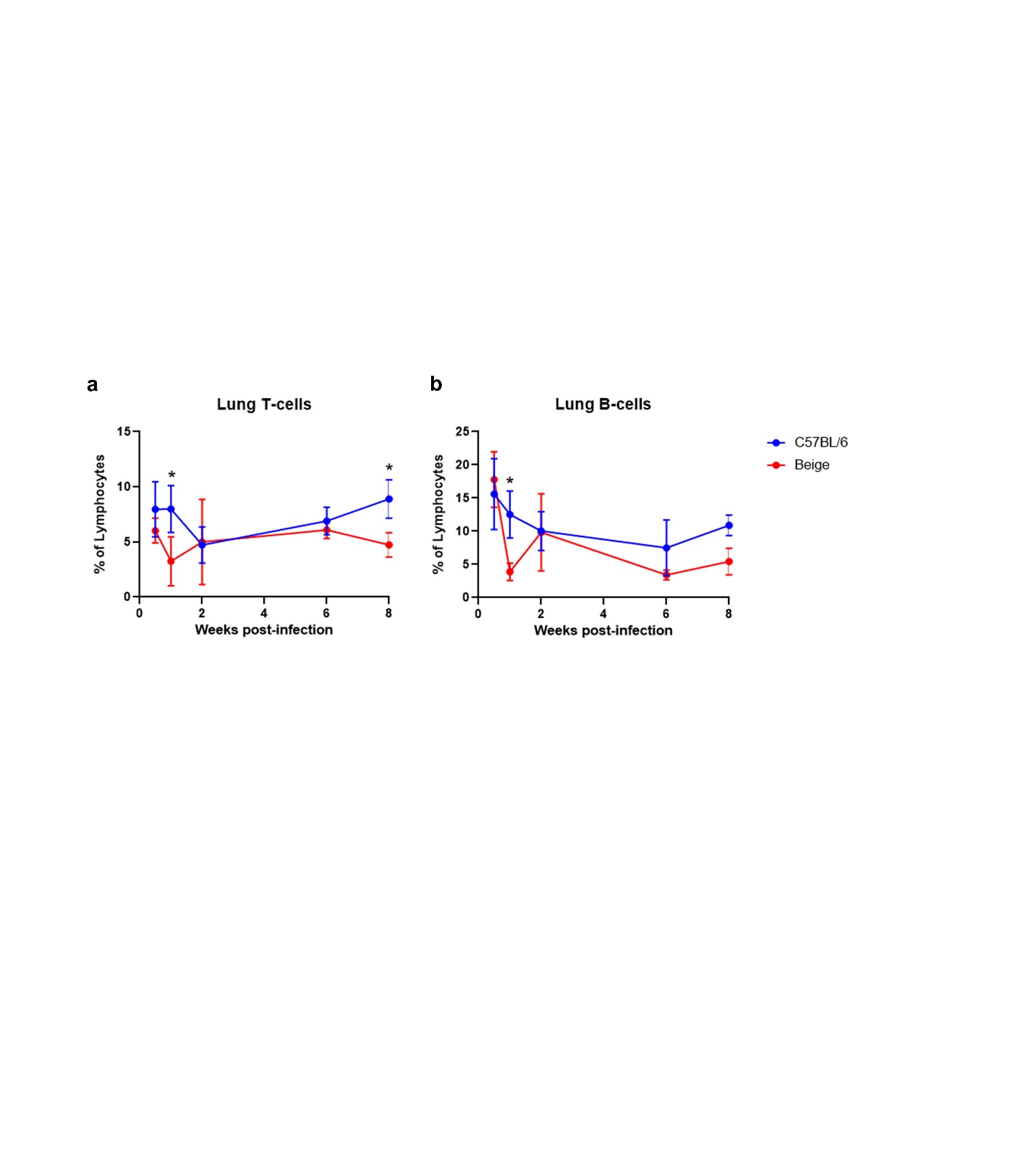


| **Supplementary Table 1: Low emergence of rough morphotype CFU *in vitro*** | | | | | | |
| --- | --- | --- | --- | --- | --- | --- |
| **Replicate** | **1** | | **2** | | **3** | |
| **Week** | **R** | **S** | **R** | **S** | **R** | **S** |
| 0 | 0 | > 300 | 0 | > 300 | 0 | > 300 |
| 1 | 0 | > 300 | 0 | > 300 | 0 | > 300 |
| 2 | 0 | > 200 | 0 | > 200 | 0 | > 200 |
| 3 | 0 | > 300 | 1 | > 300 | 0 | 168 |
| 5 | 13 | 165 | 0 | 250 | 7 | 219 |
| *M. avium* 2-151 smt infection stock (smooth phenotype, S) cultured *in vitro* and diluted weekly and plated to observe outgrowth or transition to rough colonies (R). CFU data is representative of two independent experiments. | | | | | | |

| **Supplementary Table 2: Relative morphotype ratios are preserved *in vitro*** | | | | | | |
| --- | --- | --- | --- | --- | --- | --- |
| **Replicate** | **1** | | **2** | | **3** | |
| **Week** | **R** | **S** | **R** | **S** | **R** | **S** |
| 1 | 38 | 62 | 19 | 81 | 92 | 8 |
| 2 | 5 | 95 | 12 | 88 | 98 | 2 |
| 3 | 5 | 95 | 16 | 84 | 94 | 6 |
| 5 | 13 | 87 | 30 | 70 | 96 | 4 |
| Standing culture of *M. avium* 2-151 smt smooth (S) and rough (R) colonies were generated from *ex vivo* lung homogenate plating, mixed together, diluted weekly to examine relative colony phenotypes. Data is representative of two independent experiments. | | | | | | |

| **Supplementary Table 3: Comparable drug sensitivity of morphotypes** | | |
| --- | --- | --- |
| **Compound** | **Rough  *MIC (µM)** | **Smooth  *MIC (µM)** |
| Ethambutol | 9.7 | 13 |
| Azithromycin | 12.3 | 24 |
| Clarithromycin | < 0.4 | < 0.4 |
| Linezolid | > 200 | > 200 |
| Amikacin | 10 | >20 |
| Rifampicin | < 0.4 | < 0.4 |
| Streptomycin | 4.1 | 9.6 |
| *µM concentrations established by optical density assay. | | |

| **Supplemental Table 4: Predicted MCHII binding epitopes for Mtb Rv3619 and *M. avium* homolog** | | | |
| --- | --- | --- | --- |
| **Mtb** | | ***M. avium*** | |
| **Peptide** | **Adjusted rank** | **Peptide** | **Adjusted rank** |
| FQVIYEQANAHGQKV | 4.95 | FQVIYEQANAHGQKV | 4.95 |
| RNFQVIYEQANAHGQ | 5.15 | RNFQVIYEQANAHGQ | 5.15 |
| NFQVIYEQANAHGQK | 5.25 | NFQVIYEQANAHGQK | 5.25 |
| TASDFWGGAGSAACQ | 6.35 | HGQKVQTAGSNMAST | 5.85 |
| ASDFWGGAGSAACQG | 6.4 | AHGQKVQTAGSNMAS | 6.05 |
| LTASDFWGGAGSAAC | 6.4 | GQKVQTAGSNMASTD | 6.25 |
| SDFWGGAGSAACQGF | 7.05 | QKVQTAGSNMASTDS | 6.6 |
| QVIYEQANAHGQKVQ | 7.3 | QVIYEQANAHGQKVQ | 7.3 |
| VIYEQANAHGQKVQA | 8.45 | LAAGDFWGGAGSVAC | 7.75 |
| GRNFQVIYEQANAHG | 8.55 | AAGDFWGGAGSVACQ | 7.85 |
| AHGQKVQAAGNNMAQ | 10.6 | AGDFWGGAGSVACQE | 8.05 |
| HGQKVQAAGNNMAQT | 10.75 | NAHGQKVQTAGSNMA | 8.45 |
| DFWGGAGSAACQGFI | 11 | GRNFQVIYEQANAHG | 8.55 |
| VLTASDFWGGAGSAA | 11.5 | VIYEQANAHGQKVQT | 9 |
| GQKVQAAGNNMAQTD | 11.6 | VQTAGSNMASTDSAV | 9.7 |
| LGRNFQVIYEQANAH | 11.6 | AHGALIRAQAASLEA | 10.15 |
| QKVQAAGNNMAQTDS | 12.25 | GDFWGGAGSVACQEF | 10.35 |
| NAHGQKVQAAGNNMA | 12.95 | KVQTAGSNMASTDSA | 11 |
| AHGAMIRAQAGSLEA | 14.5 | GALIRAQAASLEAEH | 11.5 |
| HGAMIRAQAGSLEAE | 16 | HGALIRAQAASLEAE | 11.5 |
| GAMIRAQAGSLEAEH | 16.5 | LGRNFQVIYEQANAH | 11.6 |
| AMIRAQAGSLEAEHQ | 17 | ALIRAQAASLEAEHQ | 13 |
| FWGGAGSAACQGFIT | 17 | DFWGGAGSVACQEFI | 13 |
| IYEQANAHGQKVQAA | 18.5 | DAHGALIRAQAASLE | 14 |
| MIRAQAGSLEAEHQA | 18.5 | VDAHGALIRAQAASL | 15 |
| VQAAGNNMAQTDSAV | 18.5 | GDVDAHGALIRAQAA | 15.5 |
| KVQAAGNNMAQTDSA | 19 | QTAGSNMASTDSAVG | 15.5 |
| GDVDAHGAMIRAQAG | 22.5 | DVDAHGALIRAQAAS | 16.5 |
| FGDVDAHGAMIRAQA | 24.5 | LIRAQAASLEAEHQA | 16.5 |
| QAAGNNMAQTDSAVG | 25 | VLAAGDFWGGAGSVA | 17 |
| VDAHGAMIRAQAGSL | 25 | TAGSNMASTDSAVGS | 17.5 |
| AGNNMAQTDSAVGSS | 25.5 | AGSNMASTDSAVGSS | 18.5 |
| ANAHGQKVQAAGNNM | 25.5 | GSNMASTDSAVGSSW | 20 |
| DVDAHGAMIRAQAGS | 25.5 | IRAQAASLEAEHQAI | 22.5 |
| QFGDVDAHGAMIRAQ | 26 | IYEQANAHGQKVQTA | 22.5 |
| AAGNNMAQTDSAVGS | 26.5 | FGDVDAHGALIRAQA | 24.5 |
| GNNMAQTDSAVGSSW | 26.5 | FWGGAGSVACQEFIT | 25.5 |
| IRAQAGSLEAEHQAI | 27 | QFGDVDAHGALIRAQ | 25.5 |
| MTINYQFGDVDAHGA | 27.5 | ANAHGQKVQTAGSNM | 26.5 |
| YEQANAHGQKVQAAG | 27.5 | MSINYQFGDVDAHGA | 27.5 |
| INYQFGDVDAHGAMI | 28 | SINYQFGDVDAHGAL | 27.5 |
| TINYQFGDVDAHGAM | 28 | SNMASTDSAVGSSWA | 27.5 |
| DAHGAMIRAQAGSLE | 28.5 | INYQFGDVDAHGALI | 28 |
| NYQFGDVDAHGAMIR | 28.5 | NYQFGDVDAHGALIR | 28 |
| DVLTASDFWGGAGSA | 29.5 | YQFGDVDAHGALIRA | 30 |
| YQFGDVDAHGAMIRA | 30 | YEQANAHGQKVQTAG | 33.5 |
| NNMAQTDSAVGSSWA | 30.5 | QANAHGQKVQTAGSN | 34 |
| QANAHGQKVQAAGNN | 31.5 | RAQAASLEAEHQAII | 34.5 |
| WGGAGSAACQGFITQ | 32.5 | DVLAAGDFWGGAGSV | 35.5 |
| EQANAHGQKVQAAGN | 36 | QLGRNFQVIYEQANA | 39 |
| RAQAGSLEAEHQAII | 36.5 | EQANAHGQKVQTAGS | 45.5 |
| QLGRNFQVIYEQANA | 39 | TQLGRNFQVIYEQAN | 47 |
| EAEHQAIISDVLTAS | 44 | WGGAGSVACQEFITQ | 48.5 |
| AEHQAIISDVLTASD | 45.5 | AQAASLEAEHQAIIR | 49.5 |
| TQLGRNFQVIYEQAN | 47 |  |  |
| EHQAIISDVLTASDF | 48.5 |  |  |
| GGAGSAACQGFITQL | 48.5 |  |  |
| LEAEHQAIISDVLTA | 49 |  |  |

| **Supplemental Table 5: Predicted MCHII binding epitopes for Mtb Rv2389 and *M. avium* homolog** | | | |
| --- | --- | --- | --- |
| **Mtb** | | ***M. avium*** | |
| **Peptide** | **Adjusted rank** | **Peptide** | **Adjusted rank** |
| DSNGGVGSPAAASPQ | 0.91 | VGGFVAASMASSTGV | 1.32 |
| SNGGVGSPAAASPQQ | 1.04 | VVGGFVAASMASSTG | 1.32 |
| NGGVGSPAAASPQQQ | 1.3 | VVVGGFVAASMASST | 1.34 |
| WDSNGGVGSPAAASP | 1.65 | TWARYGGVGNPAAAS | 1.35 |
| TWDSNGGVGSPAAAS | 1.9 | WARYGGVGNPAAASR | 1.35 |
| GGVGSPAAASPQQQI | 2 | ARYGGVGNPAAASRE | 1.4 |
| GVGSPAAASPQQQIE | 3.9 | GGFVAASMASSTGVV | 1.47 |
| ATWDSNGGVGSPAAA | 4 | GFVAASMASSTGVVS | 2.11 |
| VGSPAAASPQQQIEV | 9.45 | SVVVGGFVAASMASS | 2.2 |
| QATWDSNGGVGSPAA | 10 | ATWARYGGVGNPAAA | 2.25 |
| NIMKTQGPGAWPKCS | 10.3 | RYGGVGNPAAASREQ | 2.5 |
| DNIMKTQGPGAWPKC | 10.95 | MASSTGVVSAEPTPN | 3.08 |
| IMKTQGPGAWPKCSS | 11.5 | YGGVGNPAAASREQQ | 3.35 |
| CESGGNWAANTGNGL | 12.8 | FVAASMASSTGVVSA | 4 |
| ADNIMKTQGPGAWPK | 12.95 | KSVVVGGFVAASMAS | 4.3 |
| MKTQGPGAWPKCSSC | 13.5 | PATWARYGGVGNPAA | 5 |
| ESGGNWAANTGNGLY | 14.5 | ASSTGVVSAEPTPNW | 5.19 |
| GSPAAASPQQQIEVA | 14.8 | VAASMASSTGVVSAE | 5.35 |
| SGGNWAANTGNGLYG | 15.5 | SSTGVVSAEPTPNWD | 6.24 |
| GGNWAANTGNGLYGG | 18 | STGVVSAEPTPNWDA | 6.24 |
| TVFIETAVVATMFVA | 18 | VKSVVVGGFVAASMA | 6.3 |
| CSQGDAPLGSLTHIL | 18.5 | TGVVSAEPTPNWDAI | 6.74 |
| TGNGLYGGLQISQAT | 18.5 | GGVGNPAAASREQQI | 8.95 |
| GNGLYGGLQISQATW | 19 | AASMASSTGVVSAEP | 9.8 |
| SQGDAPLGSLTHILT | 19.5 | LPIGWYSHPAQGIKQ | 10 |
| GNWAANTGNGLYGGL | 20 | GLPIGWYSHPAQGIK | 10.3 |
| IVCTVFIETAVVATM | 20 | GVVSAEPTPNWDAIA | 10.85 |
| KTQGPGAWPKCSSCS | 20.5 | PIGWYSHPAQGIKQI | 10.85 |
| SCSQGDAPLGSLTHI | 20.5 | ASMASSTGVVSAEPT | 11.5 |
| VCTVFIETAVVATMF | 20.5 | SGLPIGWYSHPAQGI | 12 |
| CTVFIETAVVATMFV | 21 | SMASSTGVVSAEPTP | 12.2 |
| SSCSQGDAPLGSLTH | 21.5 | LVKSVVVGGFVAASM | 12.5 |
| FVALLGLSTISSKAD | 22 | IGWYSHPAQGIKQII | 13 |
| MFVALLGLSTISSKA | 22.5 | GVGNPAAASREQQIA | 13.5 |
| RIVCTVFIETAVVAT | 22.5 | VVSAEPTPNWDAIAQ | 13.9 |
| VFIETAVVATMFVAL | 22.5 | GEYGGLQFKPATWAR | 14.5 |
| SQATWDSNGGVGSPA | 23 | EYGGLQFKPATWARY | 16 |
| TQGPGAWPKCSSCSQ | 23 | LLVKSVVVGGFVAAS | 16.5 |
| GLYGGLQISQATWDS | 23.5 | ASREQQIAVANRVFA | 17.5 |
| NGLYGGLQISQATWD | 23.5 | YGGLQFKPATWARYG | 17.5 |
| NTGNGLYGGLQISQA | 23.5 | NGEYGGLQFKPATWA | 18 |
| AQCESGGNWAANTGN | 24 | QSGLPIGWYSHPAQG | 18 |
| QCESGGNWAANTGNG | 24 | SREQQIAVANRVFAE | 18 |
| QGDAPLGSLTHILTF | 25 | AASREQQIAVANRVF | 19 |
| ATMFVALLGLSTISS | 25.5 | REQQIAVANRVFAEE | 19.5 |
| TMFVALLGLSTISSK | 25.5 | KPATWARYGGVGNPA | 20.5 |
| FIETAVVATMFVALL | 26.5 | GWYSHPAQGIKQIIN | 21 |
| MTPGLLTTAGAGRPR | 27 | AAASREQQIAVANRV | 22.5 |
| VALLGLSTISSKADD | 27 | GGLQFKPATWARYGG | 23.5 |
| ALLGLSTISSKADDI | 27.5 | CESGGNWHANTGNGE | 24 |
| TPGLLTTAGAGRPRD | 29 | IKQIINGLIQAAVPR | 24 |
| VATMFVALLGLSTIS | 29 | AQSGLPIGWYSHPAQ | 24.5 |
| LLGLSTISSKADDID | 30.5 | ESGGNWHANTGNGEY | 24.5 |
| VADNIMKTQGPGAWP | 30.5 | SGGNWHANTGNGEYG | 24.5 |
| VVATMFVALLGLSTI | 31.5 | GNGEYGGLQFKPATW | 26 |
| PGLLTTAGAGRPRDR | 32 | WYSHPAQGIKQIING | 26.5 |
| AVVATMFVALLGLST | 32.5 | EQQIAVANRVFAEEG | 28.5 |
| GDAPLGSLTHILTFL | 34 | GGNWHANTGNGEYGG | 29 |
| LYGGLQISQATWDSN | 34 | QQIAVANRVFAEEGV | 29 |
| GLLTTAGAGRPRDRC | 34.5 | GNWHANTGNGEYGGL | 30 |
| EVADNIMKTQGPGAW | 35 | TGNGEYGGLQFKPAT | 30 |
| CSSCSQGDAPLGSLT | 37 | GLQFKPATWARYGGV | 31 |
| THILTFLAAETGGCS | 37 | VGNPAAASREQQIAV | 31.5 |
| LTHILTFLAAETGGC | 38 | KLLVKSVVVGGFVAA | 32.5 |
| HILTFLAAETGGCSG | 38.5 | FKPATWARYGGVGNP | 33 |
| ANTGNGLYGGLQISQ | 39 | GIKQIINGLIQAAVP | 34.5 |
| SLTHILTFLAAETGG | 39 | QGIKQIINGLIQAAV | 35.5 |
| DAPLGSLTHILTFLA | 39.5 | PWPKCGAQSGLPIGW | 36 |
| SPAAASPQQQIEVAD | 39.5 | EPWPKCGAQSGLPIG | 36.5 |
| GSLTHILTFLAAETG | 40 | KCGAQSGLPIGWYSH | 37.5 |
| IETAVVATMFVALLG | 40.5 | QIAVANRVFAEEGVE | 37.5 |
| AANTGNGLYGGLQIS | 41.5 | WPKCGAQSGLPIGWY | 38.5 |
| ILTFLAAETGGCSGS | 42 | GAQSGLPIGWYSHPA | 39 |
| QGPGAWPKCSSCSQG | 42.5 | LQFKPATWARYGGVG | 39 |
| YGGLQISQATWDSNG | 42.5 | PKCGAQSGLPIGWYS | 39 |
| IAQCESGGNWAANTG | 43 | NPAAASREQQIAVAN | 39.5 |
| LLTTAGAGRPRDRCA | 43 | QFKPATWARYGGVGN | 40 |
| NWAANTGNGLYGGLQ | 43 | PAAASREQQIAVANR | 40.5 |
| AIAQCESGGNWAANT | 44 | RVFAEEGVEPWPKCG | 41 |
| ARIVCTVFIETAVVA | 44 | ANRVFAEEGVEPWPK | 41.5 |
| LGLSTISSKADDIDW | 44 | QCESGGNWHANTGNG | 42 |
| ETAVVATMFVALLGL | 45 | AQGIKQIINGLIQAA | 42.5 |
| GPGAWPKCSSCSQGD | 45 | AVANRVFAEEGVEPW | 42.5 |
| QISQATWDSNGGVGS | 45.5 | NRVFAEEGVEPWPKC | 42.5 |
| KCSSCSQGDAPLGSL | 46 | GNPAAASREQQIAVA | 43 |
| LTFLAAETGGCSGSR | 47 | NTGNGEYGGLQFKPA | 43 |
| TAVVATMFVALLGLS | 47.5 | VEPWPKCGAQSGLPI | 43.5 |
| ISQATWDSNGGVGSP | 48 | AQCESGGNWHANTGN | 44 |
| WAANTGNGLYGGLQI | 48.5 | YSHPAQGIKQIINGL | 46 |
| DAIAQCESGGNWAAN | 49 | CGAQSGLPIGWYSHP | 47.5 |
|  |  | EEGVEPWPKCGAQSG | 47.5 |
|  |  | FAEEGVEPWPKCGAQ | 48 |
|  |  | VFAEEGVEPWPKCGA | 49 |
|  |  | VANRVFAEEGVEPWP | 50 |

| **Supplemental Table 6: Predicted MCHII binding epitopes for Mtb Rv3478 and *M. avium* homolog** | | | |
| --- | --- | --- | --- |
| **Mtb** | | ***M. avium*** | |
| **Peptide** | **Adjusted rank** | **Peptide** | **Adjusted rank** |
| AEAMYGYAATAATAT | 0.04 | AAAMYMYAGASAAAS | 0.01 |
| AMYGYAATAATATEA | 0.04 | AAMYMYAGASAAASA | 0.01 |
| EAMYGYAATAATATE | 0.04 | AMYMYAGASAAASAL | 0.01 |
| MYGYAATAATATEAL | 0.04 | DAAAMYMYAGASAAA | 0.01 |
| YGYAATAATATEALL | 0.04 | MYMYAGASAAASALT | 0.01 |
| HSMLKGLAPAAAQAV | 0.18 | QDAAAMYMYAGASAA | 0.01 |
| GYAATAATATEALLP | 0.19 | YMYAGASAAASALTP | 0.01 |
| SMLKGLAPAAAQAVE | 0.23 | AAYEAAFAMTVPPPV | 0.04 |
| YAATAATATEALLPF | 0.23 | AYEAAFAMTVPPPVI | 0.07 |
| ETAYRLTVPPPVIAE | 0.24 | AAAAAYEAAFAMTVP | 0.08 |
| YETAYRLTVPPPVIA | 0.24 | RAAAAAYEAAFAMTV | 0.08 |
| DAEAMYGYAATAATA | 0.3 | AAAAYEAAFAMTVPP | 0.1 |
| TAYRLTVPPPVIAEN | 0.3 | YEAAFAMTVPPPVIA | 0.12 |
| VPARAYAIPRTPAAG | 0.3 | AAAYEAAFAMTVPPP | 0.14 |
| MLKGLAPAAAQAVET | 0.31 | EAAFAMTVPPPVIAA | 0.15 |
| NSARMYAGPGSASLV | 0.34 | IKSLMPAASAASSAA | 0.17 |
| SARMYAGPGSASLVA | 0.34 | KSLMPAASAASSAAS | 0.18 |
| QDAEAMYGYAATAAT | 0.36 | AAFAMTVPPPVIAAN | 0.2 |
| INSARMYAGPGSASL | 0.41 | LSVPPTWAAAAPTIG | 0.24 |
| RVPARAYAIPRTPAA | 0.43 | QIKSLMPAASAASSA | 0.26 |
| ARMYAGPGSASLVAA | 0.45 | LPSTSVGASPAATAA | 0.3 |
| AYETAYRLTVPPPVI | 0.47 | PTWAAAAPTIGPTAA | 0.3 |
| LKGLAPAAAQAVETA | 0.55 | SLMPAASAASSAASG | 0.31 |
| RMYAGPGSASLVAAA | 0.55 | PPTWAAAAPTIGPTA | 0.33 |
| AYRLTVPPPVIAENR | 0.6 | NQIKSLMPAASAASS | 0.35 |
| EINSARMYAGPGSAS | 0.86 | LMPAASAASSAASGS | 0.36 |
| GSLSVPPAWAAANQA | 0.93 | MYAGASAAASALTPF | 0.36 |
| SSAGLMAAAASPYVA | 0.94 | SVPPTWAAAAPTIGP | 0.36 |
| SAGLMAAAASPYVAW | 1.02 | VPPTWAAAAPTIGPT | 0.37 |
| LSVPPAWAAANQAVT | 1.05 | ANQIKSLMPAASAAS | 0.38 |
| SLSVPPAWAAANQAV | 1.05 | PSTSVGASPAATAAG | 0.38 |
| AGLMAAAASPYVAWM | 1.06 | AFAMTVPPPVIAANR | 0.39 |
| YRLTVPPPVIAENRT | 1.12 | STSVGASPAATAAGP | 0.48 |
| AAYETAYRLTVPPPV | 1.14 | YAGASAAASALTPFT | 0.55 |
| KGLAPAAAQAVETAA | 1.21 | ALPSTSVGASPAATA | 0.56 |
| LHSMLKGLAPAAAQA | 1.25 | TSVGASPAATAAGPA | 0.62 |
| SVGSLSVPPAWAAAN | 1.25 | FAMTVPPPVIAANRA | 0.63 |
| GLMAAAASPYVAWMS | 1.36 | SVGASPAATAAGPAS | 0.66 |
| ASDLFSAASAFQSVV | 1.4 | MPAASAASSAASGSG | 0.68 |
| VGSLSVPPAWAAANQ | 1.4 | AEQTAAQARAAAAAY | 0.72 |
| VASDLFSAASAFQSV | 1.45 | QAEQTAAQARAAAAA | 0.73 |
| ALRVPARAYAIPRTP | 1.5 | VGASPAATAAGPASL | 0.73 |
| PEINSARMYAGPGSA | 1.51 | TWAAAAPTIGPTAAG | 0.78 |
| WAAANQAVTPAARAL | 1.56 | GASPAATAAGPASLL | 0.81 |
| TLHSMLKGLAPAAAQ | 1.59 | GALPSTSVGASPAAT | 0.84 |
| PAWAAANQAVTPAAR | 1.65 | ALSVPPTWAAAAPTI | 0.94 |
| SVASDLFSAASAFQS | 1.65 | ASALTPFTPPQPTTN | 0.95 |
| AAANQAVTPAARALP | 1.67 | ASPAATAAGPASLLG | 0.96 |
| ASVGSLSVPPAWAAA | 1.7 | TIGPTAAGALPSTSV | 1.02 |
| LMAAAASPYVAWMSV | 1.85 | PASASMVAAAAPYVV | 1.03 |
| SVPPAWAAANQAVTP | 1.95 | PTIGPTAAGALPSTS | 1.05 |
| GLWTAVSPHLSPLSN | 2.05 | WAAAAPTIGPTAAGA | 1.05 |
| PPAWAAANQAVTPAA | 2.05 | ALTPFTPPQPTTNPA | 1.1 |
| GGLWTAVSPHLSPLS | 2.1 | SPAATAAGPASLLGA | 1.11 |
| AANQAVTPAARALPL | 2.11 | GPWLGPASASMVAAA | 1.12 |
| GSSAGLMAAAASPYV | 2.15 | SARMYAGPGSGPLLA | 1.16 |
| SDLFSAASAFQSVVW | 2.18 | ASASMVAAAAPYVVW | 1.2 |
| AWAAANQAVTPAARA | 2.25 | ARMYAGPGSGPLLAA | 1.21 |
| LRVPARAYAIPRTPA | 2.25 | AQAEQTAAQARAAAA | 1.25 |
| NTLHSMLKGLAPAAA | 2.25 | IGPTAAGALPSTSVG | 1.25 |
| ANQAVTPAARALPLT | 2.35 | NANQIKSLMPAASAA | 1.26 |
| VPPAWAAANQAVTPA | 2.35 | QTAAQARAAAAAYEA | 1.27 |
| DLFSAASAFQSVVWG | 2.36 | AAPTIGPTAAGALPS | 1.3 |
| AASVGSLSVPPAWAA | 2.55 | SASMVAAAAPYVVWM | 1.3 |
| MYAGPGSASLVAAAK | 2.65 | GAAVAQATGTAASTN | 1.33 |
| LGGLWTAVSPHLSPL | 2.7 | LTPFTPPQPTTNPAG | 1.33 |
| GLAPAAAQAVETAAE | 2.8 | SALTPFTPPQPTTNP | 1.35 |
| IGSSAGLMAAAASPY | 2.85 | TGGPWLGPASASMVA | 1.35 |
| NALRVPARAYAIPRT | 2.9 | AAVAQATGTAASTNT | 1.39 |
| NQAVTPAARALPLTS | 2.91 | GQGAAVAQATGTAAS | 1.39 |
| GINNALRVPARAYAI | 2.95 | PAASAASSAASGSGL | 1.39 |
| SKLGGLWTAVSPHLS | 3 | QGAAVAQATGTAAST | 1.39 |
| INNALRVPARAYAIP | 3.1 | GGPWLGPASASMVAA | 1.4 |
| WTAVSPHLSPLSNVS | 3.1 | RMYAGPGSGPLLAAA | 1.42 |
| KLGGLWTAVSPHLSP | 3.15 | EQTAAQARAAAAAYE | 1.48 |
| AAAASPYVAWMSVTA | 3.2 | AAAAPTIGPTAAGAL | 1.5 |
| NNALRVPARAYAIPR | 3.4 | NSARMYAGPGSGPLL | 1.51 |
| LWTAVSPHLSPLSNV | 3.5 | PWLGPASASMVAAAA | 1.52 |
| ASPYVAWMSVTAGQA | 3.6 | APTIGPTAAGALPST | 1.55 |
| MVDFGALPPEINSAR | 3.7 | ASMVAAAAPYVVWMN | 1.55 |
| GQDAEAMYGYAATAA | 3.95 | LTGGPWLGPASASMV | 1.55 |
| TAVSPHLSPLSNVSS | 3.95 | APYVVWMNTTAAQAE | 1.65 |
| SPYVAWMSVTAGQAQ | 4.05 | ALQQLASPLSSTSSM | 1.67 |
| AAASPYVAWMSVTAG | 4.3 | TALQQLASPLSSTSS | 1.67 |
| WIGSSAGLMAAAASP | 4.35 | PYVVWMNTTAAQAEQ | 1.75 |
| LAPAAAQAVETAAEN | 4.45 | PAATAAGPASLLGAP | 1.76 |
| VDFGALPPEINSARM | 4.5 | GALSVPPTWAAAAPT | 1.85 |
| SWIGSSAGLMAAAAS | 4.55 | MYAGPGSGPLLAAAA | 1.85 |
| GSWIGSSAGLMAAAA | 4.6 | AAAPTIGPTAAGALP | 1.95 |
| QAVTPAARALPLTSL | 4.6 | AAPYVVWMNTTAAQA | 1.95 |
| YAGPGSASLVAAAKM | 4.6 | INSARMYAGPGSGPL | 1.97 |
| MAAAASPYVAWMSVT | 4.65 | LQQLASPLSSTSSMS | 2.02 |
| AASPYVAWMSVTAGQ | 4.8 | PTALQQLASPLSSTS | 2.04 |
| AVTPAARALPLTSLT | 4.85 | GPTAAGALPSTSVGA | 2.05 |
| LTSLTSAAQTAPGHM | 5.05 | TPFTPPQPTTNPAGL | 2.08 |
| AVSPHLSPLSNVSSI | 5.25 | GLTGGPWLGPASASM | 2.2 |
| DSVASDLFSAASAFQ | 5.3 | AAQAEQTAAQARAAA | 2.25 |
| TVGSWIGSSAGLMAA | 5.4 | FMPRLTVVPRSPAVG | 2.25 |
| AATAATATEALLPFE | 5.5 | QARAAAAAYEAAFAM | 2.25 |
| GRAASVGSLSVPPAW | 5.5 | YAGPGSGPLLAAAAG | 2.25 |
| VGSWIGSSAGLMAAA | 5.75 | QQLASPLSSTSSMSS | 2.26 |
| PYVAWMSVTAGQAQL | 5.85 | SMVAAAAPYVVWMNT | 2.3 |
| WDSVASDLFSAASAF | 5.9 | AVAQATGTAASTNTQ | 2.36 |
| RAASVGSLSVPPAWA | 6.1 | WLGPASASMVAAAAP | 2.4 |
| LTVGSWIGSSAGLMA | 6.2 | GPASASMVAAAAPYV | 2.45 |
| PLTSLTSAAQTAPGH | 6.2 | LGGAAVSAATGRAAS | 2.45 |
| GLTVGSWIGSSAGLM | 6.55 | PTAAGALPSTSVGAS | 2.45 |
| LGRAASVGSLSVPPA | 6.7 | SLGALSVPPTWAAAA | 2.45 |
| VSPHLSPLSNVSSIA | 6.75 | AGALPSTSVGASPAA | 2.5 |
| DFGALPPEINSARMY | 6.85 | ARAAAAAYEAAFAMT | 2.56 |
| TSLTSAAQTAPGHML | 6.9 | AASAASSAASGSGLT | 2.6 |
| ALPLTSLTSAAQTAP | 7 | AGQGAAVAQATGTAA | 2.65 |
| YVAWMSVTAGQAQLT | 7.1 | GGAAVSAATGRAASL | 2.65 |
| PAARALPLTSLTSAA | 7.2 | QAVLSQVTSTVPTAL | 2.65 |
| VTPAARALPLTSLTS | 7.2 | AVLSQVTSTVPTALQ | 2.7 |
| RALPLTSLTSAAQTA | 7.3 | LGALSVPPTWAAAAP | 2.75 |
| SGLGAGVAANLGRAA | 7.35 | LGPASASMVAAAAPY | 2.75 |
| LFSAASAFQSVVWGL | 7.45 | VLSQVTSTVPTALQQ | 2.75 |
| ATNLLGQNTPAIEAN | 7.5 | GLGGAAVSAATGRAA | 2.85 |
| GLGAGVAANLGRAAS | 7.75 | TAAQARAAAAAYEAA | 2.85 |
| AARALPLTSLTSAAQ | 7.8 | LSQVTSTVPTALQQL | 2.95 |
| SLTSAAQTAPGHMLG | 7.8 | TNILGQNTPAIAATE | 3.01 |
| LGAGVAANLGRAASV | 7.95 | GAAVSAATGRAASLG | 3.05 |
| TNLLGQNTPAIEANQ | 7.95 | PFTPPQPTTNPAGLA | 3.07 |
| ARALPLTSLTSAAQT | 8.1 | AAAPYVVWMNTTAAQ | 3.15 |
| TPAARALPLTSLTSA | 8.15 | VPTALQQLASPLSST | 3.2 |
| LPLTSLTSAAQTAPG | 8.2 | AAASALTPFTPPQPT | 3.3 |
| GAGVAANLGRAASVG | 8.45 | AASALTPFTPPQPTT | 3.3 |
| QLAQPAQGVVPSSKL | 8.45 | ATNILGQNTPAIAAT | 3.3 |
| QQLAQPAQGVVPSSK | 8.55 | TAAQAEQTAAQARAA | 3.35 |
| FGALPPEINSARMYA | 8.6 | AMTVPPPVIAANRAL | 3.4 |
| LQQLAQPAQGVVPSS | 8.65 | ASLGALSVPPTWAAA | 3.45 |
| SPHLSPLSNVSSIAN | 8.7 | RFMPRLTVVPRSPAV | 3.5 |
| AGVAANLGRAASVGS | 9 | AAVSAATGRAASLGA | 3.55 |
| SSKLGGLWTAVSPHL | 9 | AGASAAASALTPFTP | 3.55 |
| ALQQLAQPAQGVVPS | 9.2 | VAQATGTAASTNTQA | 3.55 |
| ATAATATEALLPFED | 9.25 | ASLLGAPLAGMARPN | 3.69 |
| AGPGSASLVAAAKMW | 9.35 | TVPTALQQLASPLSS | 3.7 |
| SLGSSGLGAGVAANL | 9.5 | AAQARAAAAAYEAAF | 3.71 |
| NLGRAASVGSLSVPP | 9.6 | YVVWMNTTAAQAEQT | 3.8 |
| LAQPAQGVVPSSKLG | 9.75 | AGLTGGPWLGPASAS | 3.85 |
| QAQLTAAQVRVAAAA | 9.75 | AAAAPYVVWMNTTAA | 4 |
| AQLTAAQVRVAAAAY | 10 | SNANQIKSLMPAASA | 4 |
| SSLGSSGLGAGVAAN | 10 | AAGALPSTSVGASPA | 4.05 |
| TNTLHSMLKGLAPAA | 10.15 | ILGQNTPAIAATEAQ | 4.12 |
| LGSSGLGAGVAANLG | 10.25 | NILGQNTPAIAATEA | 4.2 |
| AANLGRAASVGSLSV | 10.45 | FTPPQPTTNPAGLAG | 4.22 |
| VAANLGRAASVGSLS | 10.45 | GASAAASALTPFTPP | 4.25 |
| RLTVPPPVIAENRTE | 10.6 | PAGLAGQGAAVAQAT | 4.25 |
| NLLGQNTPAIEANQA | 10.7 | QLASPLSSTSSMSSL | 4.25 |
| ETAAENGVWAMSSLG | 10.8 | AATAAGPASLLGAPL | 4.3 |
| LTAAQVRVAAAAYET | 10.8 | GPASLLGAPLAGMAR | 4.33 |
| LLGQNTPAIEANQAA | 10.95 | SLLGAPLAGMARPNQ | 4.4 |
| AAENGVWAMSSLGSQ | 11 | IATNILGQNTPAIAA | 4.45 |
| ANLGRAASVGSLSVP | 11 | MDFGALPPEINSARM | 4.45 |
| TATNLLGQNTPAIEA | 11 | SQVTSTVPTALQQLA | 4.45 |
| LTSAAQTAPGHMLGG | 11.05 | VVWMNTTAAQAEQTA | 4.45 |
| AGQAQLTAAQVRVAA | 11.15 | TGSAGLGGAAVSAAT | 4.55 |
| TAGQAQLTAAQVRVA | 11.3 | AASLGALSVPPTWAA | 4.65 |
| FSAASAFQSVVWGLT | 11.35 | IAGLTGGPWLGPASA | 4.75 |
| GQAQLTAAQVRVAAA | 11.35 | AQARAAAAAYEAAFA | 4.8 |
| VAWMSVTAGQAQLTA | 11.35 | AGLAGQGAAVAQATG | 4.9 |
| WMSVTAGQAQLTAAQ | 11.7 | VGWISSALLSNANQI | 4.95 |
| TAAQVRVAAAAYETA | 11.75 | ASAASSAASGSGLTG | 5 |
| AWMSVTAGQAQLTAA | 11.8 | SSVGWISSALLSNAN | 5.1 |
| AAANQLMNNVPQALQ | 11.9 | SVGWISSALLSNANQ | 5.1 |
| TAAANQLMNNVPQAL | 12.1 | AQATGTAASTNTQAV | 5.15 |
| AAQVRVAAAAYETAY | 12.2 | AVSAATGRAASLGAL | 5.3 |
| GSSGLGAGVAANLGR | 12.2 | GSAGLGGAAVSAATG | 5.6 |
| ANQLMNNVPQALQQL | 12.35 | MVAAAAPYVVWMNTT | 5.6 |
| AANQLMNNVPQALQQ | 12.4 | GWISSALLSNANQIK | 5.65 |
| SSGLGAGVAANLGRA | 12.4 | LAGQGAAVAQATGTA | 5.65 |
| GVAANLGRAASVGSL | 12.5 | TAAGALPSTSVGASP | 5.65 |
| PQALQQLAQPAQGVV | 12.5 | AGPASLLGAPLAGMA | 5.7 |
| TAAENGVWAMSSLGS | 12.5 | STGSAGLGGAAVSAA | 5.8 |
| VWGLTVGSWIGSSAG | 12.5 | AQDAAAMYMYAGASA | 6 |
| WGLTVGSWIGSSAGL | 12.5 | EINSARMYAGPGSGP | 6 |
| LGHSVNAGSGINNAL | 12.6 | LSNANQIKSLMPAAS | 6 |
| AQVRVAAAAYETAYR | 12.7 | SAGLGGAAVSAATGR | 6.05 |
| PLGHSVNAGSGINNA | 12.7 | PEINSARMYAGPGSG | 6.1 |
| LTATNLLGQNTPAIE | 13 | LLGAPLAGMARPNQG | 6.2 |
| MSVTAGQAQLTAAQV | 13 | WISSALLSNANQIKS | 6.25 |
| QALQQLAQPAQGVVP | 13 | SAAASALTPFTPPQP | 6.3 |
| NQLMNNVPQALQQLA | 13.45 | LSSLSSSLSPLSTGM | 6.35 |
| GPGSASLVAAAKMWD | 13.5 | ATAAGPASLLGAPLA | 6.45 |
| WGQDAEAMYGYAATA | 13.5 | PASLLGAPLAGMARP | 6.46 |
| PSSKLGGLWTAVSPH | 14 | LIATNILGQNTPAIA | 6.55 |
| VPQALQQLAQPAQGV | 14 | ASAAASALTPFTPPQ | 6.6 |
| QLTAAQVRVAAAAYE | 14.1 | GRAASLGALSVPPTW | 6.6 |
| GHSVNAGSGINNALR | 14.25 | VSAATGRAASLGALS | 6.7 |
| AQPAQGVVPSSKLGG | 14.5 | AGLGGAAVSAATGRA | 6.75 |
| GLPLGHSVNAGSGIN | 14.5 | GLAGQGAAVAQATGT | 6.75 |
| PHLSPLSNVSSIANN | 14.5 | DFGALPPEINSARMY | 6.85 |
| QVRVAAAAYETAYRL | 14.5 | TGRAASLGALSVPPT | 7.05 |
| SVTAGQAQLTAAQVR | 14.5 | LASPLSSTSSMSSLS | 7.15 |
| VTAGQAQLTAAQVRV | 14.5 | SSLSSLSSSLSPLST | 7.15 |
| GHMLGGLPLGHSVNA | 15 | VAAAAPYVVWMNTTA | 7.15 |
| HLSPLSNVSSIANNH | 15.5 | SLSSLSSSLSPLSTG | 7.3 |
| NVPQALQQLAQPAQG | 15.5 | AGPGSGPLLAAAAGW | 7.45 |
| PGHMLGGLPLGHSVN | 15.5 | ASGLTAGLGPGAQGS | 7.45 |
| SGINNALRVPARAYA | 15.5 | SGLTAGLGPGAQGST | 7.55 |
| AENGVWAMSSLGSQL | 16 | SSLSSSLSPLSTGMS | 7.9 |
| ENGVWAMSSLGSQLG | 16 | ATGRAASLGALSVPP | 8.1 |
| LPLGHSVNAGSGINN | 16 | MTVPPPVIAANRALL | 8.15 |
| ALLPFEDAPLITNPG | 16.5 | TQAVLSQVTSTVPTA | 8.3 |
| APAAAQAVETAAENG | 16.5 | LGAPLAGMARPNQGT | 8.4 |
| HMLGGLPLGHSVNAG | 16.5 | SAATGRAASLGALSV | 8.45 |
| MLGGLPLGHSVNAGS | 17 | AAGPASLLGAPLAGM | 8.5 |
| TSAAQTAPGHMLGGL | 17 | AATGRAASLGALSVP | 8.55 |
| HSVNAGSGINNALRV | 17.5 | FGALPPEINSARMYA | 8.6 |
| LSPLSNVSSIANNHM | 17.5 | QATGTAASTNTQAVL | 8.65 |
| EALLPFEDAPLITNP | 18 | GPGSGPLLAAAAGWD | 8.75 |
| QPAQGVVPSSKLGGL | 18 | LGQNTPAIAATEAQY | 8.8 |
| VRVAAAAYETAYRLT | 18 | NPAGLAGQGAAVAQA | 8.85 |
| AVEEAIDTAAANQLM | 18.5 | RAASLGALSVPPTWA | 8.9 |
| GSGINNALRVPARAY | 18.5 | GLTAGLGPGAQGSTG | 8.95 |
| GSSLGSSGLGAGVAA | 18.5 | TAAGPASLLGAPLAG | 9 |
| LGQNTPAIEANQAAY | 18.5 | ATGTAASTNTQAVLS | 9.4 |
| TEALLPFEDAPLITN | 18.5 | DGLAADVASTATSYS | 9.65 |
| VAVEEAIDTAAANQL | 18.5 | MSSLSSLSSSLSPLS | 9.65 |
| VEEAIDTAAANQLMN | 18.5 | ASPLSSTSSMSSLSS | 9.75 |
| LGGLPLGHSVNAGSG | 19 | GLASGLTAGLGPGAQ | 10 |
| RVAAAAYETAYRLTV | 19 | QVTSTVPTALQQLAS | 10 |
| LLPFEDAPLITNPGG | 19.5 | VWMNTTAAQAEQTAA | 10.15 |
| PAAAQAVETAAENGV | 20 | WDGLAADVASTATSY | 10.45 |
| GQNTPAIEANQAAYS | 20.5 | TNPAGLAGQGAAVAQ | 10.55 |
| NGVWAMSSLGSQLGS | 20.5 | LASGLTAGLGPGAQG | 10.6 |
| PGSASLVAAAKMWDS | 20.5 | TVPPPVIAANRALLM | 10.7 |
| QLMNNVPQALQQLAQ | 20.5 | SLSSSLSPLSTGMSM | 10.85 |
| SVNAGSGINNALRVP | 20.5 | TSSVGWISSALLSNA | 10.95 |
| VETAAENGVWAMSSL | 20.5 | GAPLAGMARPNQGTA | 11 |
| EEAIDTAAANQLMNN | 21 | GLAADVASTATSYSS | 11.25 |
| LPFEDAPLITNPGGL | 21 | AGWDGLAADVASTAT | 11.5 |
| AAAYETAYRLTVPPP | 21.5 | ISSALLSNANQIKSL | 11.5 |
| AGSGINNALRVPARA | 21.5 | LTAGLGPGAQGSTGS | 11.5 |
| MWDSVASDLFSAASA | 21.5 | TTNPAGLAGQGAAVA | 11.6 |
| QNTPAIEANQAAYSQ | 21.5 | GQNTPAIAATEAQYG | 11.8 |
| SPLSNVSSIANNHMS | 21.5 | GWDGLAADVASTATS | 12 |
| EAIDTAAANQLMNNV | 22 | LAADVASTATSYSSV | 12.45 |
| NNVPQALQQLAQPAQ | 22 | GSTGSAGLGGAAVSA | 12.5 |
| AAAAYETAYRLTVPP | 22.5 | TGTAASTNTQAVLSQ | 12.8 |
| TLTATNLLGQNTPAI | 22.5 | PGSGPLLAAAAGWDG | 13 |
| APGHMLGGLPLGHSV | 23 | SAASSAASGSGLTGG | 13 |
| AVETAAENGVWAMSS | 23 | WMNTTAAQAEQTAAQ | 13 |
| GVWAMSSLGSQLGSS | 23.5 | WAQDAAAMYMYAGAS | 13.35 |
| SAAQTAPGHMLGGLP | 23.5 | AADVASTATSYSSVI | 13.5 |
| LGSSLGSSGLGAGVA | 24 | APLAGMARPNQGTAG | 13.5 |
| AQAVETAAENGVWAM | 24.5 | SMSSLSSLSSSLSPL | 13.5 |
| AVAVEEAIDTAAANQ | 24.5 | TAGLGPGAQGSTGSA | 13.5 |
| GGLPLGHSVNAGSGI | 24.5 | AQYGEMWAQDAAAMY | 14 |
| LTVPPPVIAENRTEL | 24.5 | MTSSVGWISSALLSN | 14 |
| PLSNVSSIANNHMSM | 24.5 | SSALLSNANQIKSLM | 14 |
| LMNNVPQALQQLAQP | 25 | DVASTATSYSSVIAG | 14.5 |
| MMGTGVSMTNTLHSM | 25 | MNTTAAQAEQTAAQA | 14.5 |
| MNNVPQALQQLAQPA | 25 | PLAGMARPNQGTAGD | 14.5 |
| QAVETAAENGVWAMS | 25 | ASTATSYSSVIAGLT | 14.95 |
| VAAAAYETAYRLTVP | 25 | AAGWDGLAADVASTA | 15 |
| ATEALLPFEDAPLIT | 25.5 | AASSAASGSGLTGGL | 15 |
| GSASLVAAAKMWDSV | 25.5 | GSGLTGGLASGLTAG | 15 |
| MGTGVSMTNTLHSML | 25.5 | LSSSLSPLSTGMSMT | 15 |
| NTPAIEANQAAYSQM | 25.5 | QYGEMWAQDAAAMYM | 15 |
| TAATATEALLPFEDA | 25.5 | YGEMWAQDAAAMYMY | 15 |
| TPAIEANQAAYSQMW | 25.5 | NTTAAQAEQTAAQAR | 15.5 |
| AAAQAVETAAENGVW | 26 | PLSSTSSMSSLSSLS | 15.5 |
| AAQTAPGHMLGGLPL | 26.5 | QGSTGSAGLGGAAVS | 15.5 |
| MSMMGTGVSMTNTLH | 26.5 | SGLTGGLASGLTAGL | 15.5 |
| NAGSGINNALRVPAR | 26.5 | SPLSSTSSMSSLSSL | 15.5 |
| TAPGHMLGGLPLGHS | 26.5 | ADVASTATSYSSVIA | 16 |
| GALPPEINSARMYAG | 27 | EAQYGEMWAQDAAAM | 16 |
| QAVAVEEAIDTAAAN | 27 | LSSTSSMSSLSSLSS | 16 |
| SMMGTGVSMTNTLHS | 27 | NTQAVLSQVTSTVPT | 16 |
| HMSMMGTGVSMTNTL | 27.5 | SSMSSLSSLSSSLSP | 16 |
| LSNVSSIANNHMSMM | 27.5 | STVPTALQQLASPLS | 16 |
| PAIEANQAAYSQMWG | 28 | TSSMSSLSSLSSSLS | 16 |
| QTAPGHMLGGLPLGH | 28 | TTAAQAEQTAAQARA | 16 |
| AIDTAAANQLMNNVP | 28.5 | STSSMSSLSSLSSSL | 16.5 |
| PGGLLEQAVAVEEAI | 28.5 | VASTATSYSSVIAGL | 16.5 |
| ALPPEINSARMYAGP | 29.5 | VPPPVIAANRALLMS | 16.5 |
| AQTAPGHMLGGLPLG | 29.5 | VTSTVPTALQQLASP | 16.5 |
| PFEDAPLITNPGGLL | 29.5 | QNTPAIAATEAQYGE | 16.85 |
| TATEALLPFEDAPLI | 29.5 | GTAASTNTQAVLSQV | 17 |
| AIEANQAAYSQMWGQ | 30 | SSTSSMSSLSSLSSS | 18 |
| GTGVSMTNTLHSMLK | 30.5 | LAGMARPNQGTAGDA | 18.5 |
| IDTAAANQLMNNVPQ | 30.5 | LLSNANQIKSLMPAA | 18.5 |
| PAQGVVPSSKLGGLW | 31 | SSSLSPLSTGMSMTS | 18.5 |
| NNHMSMMGTGVSMTN | 31.5 | STATSYSSVIAGLTG | 18.5 |
| DTAAANQLMNNVPQA | 32 | ASSAASGSGLTGGLA | 19 |
| NHMSMMGTGVSMTNT | 32 | GEMWAQDAAAMYMYA | 19 |
| VNAGSGINNALRVPA | 32 | SSAASGSGLTGGLAS | 19 |
| ANNHMSMMGTGVSMT | 32.5 | GLTGGLASGLTAGLG | 19.5 |
| PPEINSARMYAGPGS | 32.5 | VIAGLTGGPWLGPAS | 19.5 |
| TGVSMTNTLHSMLKG | 32.5 | AQGSTGSAGLGGAAV | 20 |
| KMWDSVASDLFSAAS | 33 | GLGPGAQGSTGSAGL | 20 |
| LITNPGGLLEQAVAV | 33 | LRFMPRLTVVPRSPA | 20 |
| ENRTELMTLTATNLL | 33.5 | PPPVIAANRALLMSL | 20 |
| GGLLEQAVAVEEAID | 33.5 | SAASGSGLTGGLASG | 20 |
| NRTELMTLTATNLLG | 33.5 | SGSGLTGGLASGLTA | 20 |
| FEDAPLITNPGGLLE | 34 | AGLGPGAQGSTGSAG | 20.5 |
| GSQLGSSLGSSGLGA | 34 | GSGPLLAAAAGWDGL | 20.5 |
| NPGGLLEQAVAVEEA | 34 | SMTSSVGWISSALLS | 20.5 |
| SASLVAAAKMWDSVA | 34 | TATSYSSVIAGLTGG | 20.5 |
| VVWGLTVGSWIGSSA | 34 | TNTQAVLSQVTSTVP | 20.5 |
| ASLVAAAKMWDSVAS | 34.5 | ADLRFMPRLTVVPRS | 21 |
| SQLGSSLGSSGLGAG | 34.5 | LTGGLASGLTAGLGP | 21 |
| AAQAVETAAENGVWA | 35 | MSMTSSVGWISSALL | 21 |
| GLLEQAVAVEEAIDT | 35 | PTTNPAGLAGQGAAV | 21 |
| IEANQAAYSQMWGQD | 35 | TPPQPTTNPAGLAGQ | 21 |
| ITNPGGLLEQAVAVE | 35.5 | DLRFMPRLTVVPRSP | 21.1 |
| SLVAAAKMWDSVASD | 35.5 | AASGSGLTGGLASGL | 21.5 |
| TNPGGLLEQAVAVEE | 35.5 | ASGSGLTGGLASGLT | 21.5 |
| VPSSKLGGLWTAVSP | 35.5 | LGPGAQGSTGSAGLG | 21.5 |
| VWAMSSLGSQLGSSL | 35.5 | SLIATNILGQNTPAI | 21.5 |
| QLGSSLGSSGLGAGV | 36.5 | TEAQYGEMWAQDAAA | 21.5 |
| RTELMTLTATNLLGQ | 36.5 | PPVIAANRALLMSLI | 22 |
| AKMWDSVASDLFSAA | 37 | PVIAANRALLMSLIA | 22 |
| LGSQLGSSLGSSGLG | 37 | AGMARPNQGTAGDAD | 22.5 |
| SLGSQLGSSLGSSGL | 37.5 | DADLRFMPRLTVVPR | 22.5 |
| QAAYSQMWGQDAEAM | 38 | GDADLRFMPRLTVVP | 22.5 |
| AAKMWDSVASDLFSA | 39 | LSPLSTGMSMTSSVG | 22.5 |
| EDAPLITNPGGLLEQ | 39 | TSTVPTALQQLASPL | 22.5 |
| SNVSSIANNHMSMMG | 39 | SLSPLSTGMSMTSSV | 23.5 |
| TELMTLTATNLLGQN | 39 | SSLSPLSTGMSMTSS | 23.5 |
| AAYSQMWGQDAEAMY | 39.5 | AAAGWDGLAADVAST | 24 |
| DAPLITNPGGLLEQA | 39.5 | EMWAQDAAAMYMYAG | 24 |
| MTNTLHSMLKGLAPA | 39.5 | GMARPNQGTAGDADL | 24 |
| EANQAAYSQMWGQDA | 40 | STNTQAVLSQVTSTV | 24 |
| LVAAAKMWDSVASDL | 40 | GMSMTSSVGWISSAL | 25 |
| NQAAYSQMWGQDAEA | 40 | LAAAAGWDGLAADVA | 25 |
| VAAAKMWDSVASDLF | 40 | SGPLLAAAAGWDGLA | 25 |
| FQSVVWGLTVGSWIG | 40.5 | TGGLASGLTAGLGPG | 25 |
| MWGQDAEAMYGYAAT | 40.5 | AAAAGWDGLAADVAS | 25.5 |
| ATATEALLPFEDAPL | 41 | TAASTNTQAVLSQVT | 25.5 |
| GVVPSSKLGGLWTAV | 41 | PPQPTTNPAGLAGQG | 26 |
| VVPSSKLGGLWTAVS | 41 | SALLSNANQIKSLMP | 26 |
| AQGVVPSSKLGGLWT | 41.5 | ALLSNANQIKSLMPA | 26.5 |
| IANNHMSMMGTGVSM | 41.5 | GGLASGLTAGLGPGA | 26.5 |
| QSVVWGLTVGSWIGS | 41.5 | NTPAIAATEAQYGEM | 26.5 |
| SAASAFQSVVWGLTV | 41.5 | GALPPEINSARMYAG | 27 |
| AENRTELMTLTATNL | 42 | GPGAQGSTGSAGLGG | 27 |
| APLITNPGGLLEQAV | 42 | SPLSTGMSMTSSVGW | 27 |
| EQAVAVEEAIDTAAA | 42 | ATSYSSVIAGLTGGP | 27.5 |
| MTLTATNLLGQNTPA | 42.5 | GAQGSTGSAGLGGAA | 28 |
| QMWGQDAEAMYGYAA | 42.5 | PGAQGSTGSAGLGGA | 28 |
| PLITNPGGLLEQAVA | 43 | ALPPEINSARMYAGP | 29.5 |
| AAAKMWDSVASDLFS | 43.5 | LSTGMSMTSSVGWIS | 30 |
| GVSMTNTLHSMLKGL | 43.5 | QPTTNPAGLAGQGAA | 30.5 |
| SSLGSQLGSSLGSSG | 44 | ANRALLMSLIATNIL | 31.5 |
| SVVWGLTVGSWIGSS | 44 | PLSTGMSMTSSVGWI | 31.5 |
| ANQAAYSQMWGQDAE | 44.5 | AASTNTQAVLSQVTS | 32 |
| MSSLGSQLGSSLGSS | 44.5 | PPEINSARMYAGPGS | 32.5 |
| SIANNHMSMMGTGVS | 44.5 | ASTNTQAVLSQVTST | 33.5 |
| TVPPPVIAENRTELM | 45 | NRALLMSLIATNILG | 33.5 |
| WAMSSLGSQLGSSLG | 45 | TSYSSVIAGLTGGPW | 34 |
| LEQAVAVEEAIDTAA | 45.5 | PQPTTNPAGLAGQGA | 34.5 |
| AMSSLGSQLGSSLGS | 46 | STGMSMTSSVGWISS | 34.5 |
| LLEQAVAVEEAIDTA | 46 | TGMSMTSSVGWISSA | 34.5 |
| NVSSIANNHMSMMGT | 46 | AANRALLMSLIATNI | 35 |
| AFQSVVWGLTVGSWI | 46.5 | AGDADLRFMPRLTVV | 35.5 |
| LPPEINSARMYAGPG | 46.5 | VIAANRALLMSLIAT | 35.5 |
| QGVVPSSKLGGLWTA | 46.5 | RALLMSLIATNILGQ | 36 |
| AATATEALLPFEDAP | 48 | SVIAGLTGGPWLGPA | 36 |
| AYSQMWGQDAEAMYG | 48.5 | YSSVIAGLTGGPWLG | 36.5 |
| LMTLTATNLLGQNTP | 48.5 | MARPNQGTAGDADLR | 37.5 |
| ASAFQSVVWGLTVGS | 49.5 | MWAQDAAAMYMYAGA | 37.5 |
| ELMTLTATNLLGQNT | 50 | TPAIAATEAQYGEMW | 38.5 |
|  |  | IAANRALLMSLIATN | 40 |
|  |  | ATEAQYGEMWAQDAA | 40.5 |
|  |  | LLAAAAGWDGLAADV | 40.5 |
|  |  | SSVIAGLTGGPWLGP | 40.5 |
|  |  | PLLAAAAGWDGLAAD | 41 |
|  |  | GPLLAAAAGWDGLAA | 42 |
|  |  | ALLMSLIATNILGQN | 43 |
|  |  | ARPNQGTAGDADLRF | 43.5 |
|  |  | AATEAQYGEMWAQDA | 45.5 |
|  |  | LPPEINSARMYAGPG | 46.5 |
|  |  | MSLIATNILGQNTPA | 47 |
|  |  | SYSSVIAGLTGGPWL | 47 |

| **Supplemental Table 7: Predicted MCHII binding epitopes for Mtb Rv1886 and *M. avium* homolog** | | | |
| --- | --- | --- | --- |
| **Mtb** | | ***M. avium*** | |
| **Peptide** | **Adjusted rank** | **Peptide** | **Adjusted rank** |
| HPQQFIYAGSLSALL | 0.11 | HPDQFIYAGSLSALL | 0.15 |
| PQQFIYAGSLSALLD | 0.15 | PDQFIYAGSLSALLD | 0.18 |
| QQFIYAGSLSALLDP | 0.17 | DQFIYAGSLSALLDP | 0.2 |
| YHPQQFIYAGSLSAL | 0.2 | NHPDQFIYAGSLSAL | 0.24 |
| AYHPQQFIYAGSLSA | 0.31 | QFIYAGSLSALLDPS | 0.48 |
| KFQDAYNAAGGHNAV | 0.46 | VNHPDQFIYAGSLSA | 0.49 |
| FQDAYNAAGGHNAVF | 0.47 | FIYAGSLSALLDPSQ | 1.07 |
| QFIYAGSLSALLDPS | 0.48 | GLIGLAGGAATANAF | 1.2 |
| QDAYNAAGGHNAVFN | 0.63 | LIGLAGGAATANAFS | 1.25 |
| LKFQDAYNAAGGHNA | 0.73 | IGLAGGAATANAFSR | 1.55 |
| DAYNAAGGHNAVFNF | 0.96 | PGLIGLAGGAATANA | 1.6 |
| FIYAGSLSALLDPSQ | 1.07 | AVGISMSGSSAMILA | 2.25 |
| LSANRAVKPTGSAAI | 1.12 | FEWYYQSGLSVIMPV | 2.25 |
| ANRAVKPTGSAAIGL | 1.43 | LPGLIGLAGGAATAN | 2.25 |
| SANRAVKPTGSAAIG | 1.43 | PAFEWYYQSGLSVIM | 2.25 |
| NRAVKPTGSAAIGLS | 1.57 | AAVGISMSGSSAMIL | 2.26 |
| IGLSMAGSSAMILAA | 1.67 | VGISMSGSSAMILAV | 2.27 |
| AIGLSMAGSSAMILA | 1.7 | AFEWYYQSGLSVIMP | 2.35 |
| AAIGLSMAGSSAMIL | 1.75 | SAAVGISMSGSSAMI | 2.55 |
| RAVKPTGSAAIGLSM | 1.98 | GLAGGAATANAFSRP | 3.1 |
| SAAIGLSMAGSSAMI | 2.05 | GSAAVGISMSGSSAM | 3.1 |
| PAFEWYYQSGLSIVM | 2.15 | KFQDAYNGAGGHNAV | 3.1 |
| FEWYYQSGLSIVMPV | 2.2 | FQDAYNGAGGHNAVF | 3.2 |
| AFEWYYQSGLSIVMP | 2.25 | EWYYQSGLSVIMPVG | 3.5 |
| AYNAAGGHNAVFNFP | 2.5 | GRRLLVGAAAAVTLP | 3.5 |
| GSAAIGLSMAGSSAM | 2.65 | WGRRLLVGAAAAVTL | 3.55 |
| EWYYQSGLSIVMPVG | 3.1 | TPAFEWYYQSGLSVI | 3.7 |
| GLVGLAGGAATAGAF | 3.5 | GTHSWEYWGAQLNAM | 3.85 |
| GTHSWEYWGAQLNAM | 3.85 | THSWEYWGAQLNAMK | 3.86 |
| THSWEYWGAQLNAMK | 3.86 | LKFQDAYNGAGGHNA | 4 |
| LVGLAGGAATAGAFS | 3.9 | QDAYNGAGGHNAVFN | 4.05 |
| PGLVGLAGGAATAGA | 3.9 | RRLLVGAAAAVTLPG | 4.05 |
| TPAFEWYYQSGLSIV | 3.9 | LAGGAATANAFSRPG | 4.1 |
| HNAVFNFPPNGTHSW | 4 | HSWEYWGAQLNAMKP | 4.17 |
| GLSMAGSSAMILAAY | 4.25 | AWGRRLLVGAAAAVT | 4.2 |
| HSWEYWGAQLNAMKG | 4.3 | DAYNGAGGHNAVFNF | 5 |
| AVKPTGSAAIGLSMA | 4.4 | VQFQSGGNGSPAVYL | 5.6 |
| LPGLVGLAGGAATAG | 4.6 | AVFNFNANGTHSWEY | 5.7 |
| AATAGAFSRPGLPVE | 5 | SWEYWGAQLNAMKPD | 5.75 |
| KVQFQSGGNNSPAVY | 5 | RLLVGAAAAVTLPGL | 5.8 |
| IKVQFQSGGNNSPAV | 5.35 | KVQFQSGGNGSPAVY | 5.9 |
| VGLAGGAATAGAFSR | 5.4 | HNAVFNFNANGTHSW | 5.95 |
| NAVFNFPPNGTHSWE | 5.45 | NAVFNFNANGTHSWE | 6.05 |
| VQFQSGGNNSPAVYL | 5.6 | GGAATANAFSRPGLP | 6.2 |
| GHNAVFNFPPNGTHS | 5.65 | WYYQSGLSVIMPVGG | 6.2 |
| GGAATAGAFSRPGLP | 5.8 | QFQSGGNGSPAVYLL | 6.35 |
| WLSANRAVKPTGSAA | 5.85 | NGTHSWEYWGAQLNA | 6.75 |
| WYYQSGLSIVMPVGG | 5.85 | RAWGRRLLVGAAAAV | 6.8 |
| WGRRLMIGTAAAVVL | 5.9 | AATANAFSRPGLPVE | 6.85 |
| GRRLMIGTAAAVVLP | 6.1 | GISMSGSSAMILAVN | 6.85 |
| LSMAGSSAMILAAYH | 6.25 | ATANAFSRPGLPVEY | 7.2 |
| AWGRRLMIGTAAAVV | 6.65 | TANAFSRPGLPVEYL | 7.3 |
| RRLMIGTAAAVVLPG | 6.65 | LPVEYLQVPSAGMGR | 7.35 |
| NGTHSWEYWGAQLNA | 6.75 | IKVQFQSGGNGSPAV | 7.4 |
| ATAGAFSRPGLPVEY | 6.8 | FQSGGNGSPAVYLLD | 7.45 |
| SWEYWGAQLNAMKGD | 6.8 | TLPGLIGLAGGAATA | 7.55 |
| LPVEYLQVPSPSMGR | 6.85 | VFNFNANGTHSWEYW | 7.65 |
| TAGAFSRPGLPVEYL | 6.85 | WEYWGAQLNAMKPDL | 7.75 |
| GGHNAVFNFPPNGTH | 6.95 | ANGTHSWEYWGAQLN | 8.45 |
| VLPGLVGLAGGAATA | 6.95 | ISMSGSSAMILAVNH | 8.45 |
| AVFNFPPNGTHSWEY | 7 | LLVGAAAAVTLPGLI | 8.65 |
| VEYLQVPSPSMGRDI | 7 | GLPVEYLQVPSAGMG | 8.85 |
| PVEYLQVPSPSMGRD | 7.15 | SFYADWYQPACGKAG | 9.2 |
| GAATAGAFSRPGLPV | 7.3 | GAATANAFSRPGLPV | 9.35 |
| GLPVEYLQVPSPSMG | 7.35 | FYADWYQPACGKAGC | 9.4 |
| GLAGGAATAGAFSRP | 7.4 | AYNGAGGHNAVFNFN | 9.55 |
| QFQSGGNNSPAVYLL | 7.7 | FNFNANGTHSWEYWG | 9.75 |
| YNAAGGHNAVFNFPP | 8.05 | PGLPVEYLQVPSAGM | 9.9 |
| RAWGRRLMIGTAAAV | 8.3 | KGVKRTGSAAVGISM | 10.1 |
| SFYSDWYSPACGKAG | 8.4 | NKGVKRTGSAAVGIS | 10.4 |
| PNGTHSWEYWGAQLN | 8.5 | ANAFSRPGLPVEYLQ | 10.45 |
| VKPTGSAAIGLSMAG | 8.55 | NTPAFEWYYQSGLSV | 10.5 |
| ELPQWLSANRAVKPT | 8.8 | PVEYLQVPSAGMGRD | 10.75 |
| FYSDWYSPACGKAGC | 9.2 | YADWYQPACGKAGCS | 10.85 |
| SELPQWLSANRAVKP | 9.25 | ADWYQPACGKAGCST | 11.25 |
| WEYWGAQLNAMKGDL | 9.3 | AGGAATANAFSRPGL | 11.5 |
| TSELPQWLSANRAVK | 9.65 | SNKGVKRTGSAAVGI | 11.5 |
| LAGGAATAGAFSRPG | 9.7 | VEYLQVPSAGMGRDI | 11.5 |
| YSDWYSPACGKAGCQ | 10 | VRAWGRRLLVGAAAA | 11.5 |
| FQSGGNNSPAVYLLD | 10.05 | RLWVYCGNGTPSELG | 12.8 |
| AGAFSRPGLPVEYLQ | 10.1 | RPGLPVEYLQVPSAG | 12.85 |
| LTSELPQWLSANRAV | 10.15 | ALLDPSQGMGPSLIG | 13 |
| AGGHNAVFNFPPNGT | 10.7 | LDPSQGMGPSLIGLA | 13 |
| PQWLSANRAVKPTGS | 10.8 | LVGAAAAVTLPGLIG | 13 |
| NTPAFEWYYQSGLSI | 11.05 | TRLWVYCGNGTPSEL | 13 |
| DIKVQFQSGGNNSPA | 11.25 | QSGGNGSPAVYLLDG | 13.45 |
| AGGAATAGAFSRPGL | 11.45 | LLDPSQGMGPSLIGL | 13.5 |
| PGLPVEYLQVPSPSM | 11.45 | YYQSGLSVIMPVGGQ | 13.5 |
| SDWYSPACGKAGCQT | 11.5 | YKADAMWGPSSDPAW | 13.7 |
| RLMIGTAAAVVLPGL | 11.55 | ASNKGVKRTGSAAVG | 14 |
| YYQSGLSIVMPVGGQ | 12 | DWYQPACGKAGCSTY | 14 |
| QWLSANRAVKPTGSA | 12.2 | SALLDPSQGMGPSLI | 14 |
| EYLQVPSPSMGRDIK | 12.5 | NTRLWVYCGNGTPSE | 14.1 |
| ALLDPSQGMGPSLIG | 13 | GHNAVFNFNANGTHS | 14.15 |
| FLTSELPQWLSANRA | 13 | NAFSRPGLPVEYLQV | 14.35 |
| LDPSQGMGPSLIGLA | 13 | DPSQGMGPSLIGLAM | 14.5 |
| GAFSRPGLPVEYLQV | 13.25 | GVKRTGSAAVGISMS | 14.5 |
| LLDPSQGMGPSLIGL | 13.5 | KADAMWGPSSDPAWQ | 15 |
| LPQWLSANRAVKPTG | 13.5 | YNGAGGHNAVFNFNA | 15.25 |
| AAGGHNAVFNFPPNG | 14 | LWVYCGNGTPSELGG | 15.5 |
| SALLDPSQGMGPSLI | 14 | GGHNAVFNFNANGTH | 16 |
| VFNFPPNGTHSWEYW | 14 | EYWGAQLNAMKPDLQ | 16.5 |
| DPSQGMGPSLIGLAM | 14.5 | STYKWETFLTSELPS | 16.5 |
| RDIKVQFQSGGNNSP | 14.5 | CSTYKWETFLTSELP | 17 |
| DWYSPACGKAGCQTY | 15 | VKRTGSAAVGISMSG | 17 |
| RLWVYCGNGTPNELG | 15.4 | LASNKGVKRTGSAAV | 17.35 |
| IRAWGRRLMIGTAAA | 16 | ADAMWGPSSDPAWQR | 17.5 |
| TRLWVYCGNGTPNEL | 16 | SRPGLPVEYLQVPSA | 17.85 |
| YKAADMWGPSSDPAW | 16 | DDYNGWDINTPAFEW | 18 |
| CQTYKWETFLTSELP | 17 | QDDYNGWDINTPAFE | 18 |
| NTRLWVYCGNGTPNE | 17 | VGAAAAVTLPGLIGL | 18 |
| QTYKWETFLTSELPQ | 17 | GYKADAMWGPSSDPA | 18.5 |
| YLQVPSPSMGRDIKV | 17.5 | IYAGSLSALLDPSQG | 18.5 |
| DDYNGWDINTPAFEW | 18 | SGGNGSPAVYLLDGL | 18.9 |
| LMIGTAAAVVLPGLV | 18 | GCSTYKWETFLTSEL | 19 |
| QDDYNGWDINTPAFE | 18 | WVYCGNGTPSELGGA | 20 |
| SSFYSDWYSPACGKA | 18 | YKWETFLTSELPSYL | 20 |
| GCQTYKWETFLTSEL | 18.5 | YWGAQLNAMKPDLQG | 20 |
| IYAGSLSALLDPSQG | 18.5 | TYKWETFLTSELPSY | 20.5 |
| KAADMWGPSSDPAWE | 18.5 | MPAEFLENFVRSSNL | 21 |
| LWVYCGNGTPNELGG | 19 | DYNGWDINTPAFEWY | 21.5 |
| FNFPPNGTHSWEYWG | 19.5 | GGYKADAMWGPSSDP | 21.5 |
| VVLPGLVGLAGGAAT | 19.5 | PAEFLENFVRSSNLK | 21.5 |
| LSIVMPVGGQSSFYS | 20.5 | TGSAAVGISMSGSSA | 21.5 |
| AADMWGPSSDPAWER | 21 | AEFLENFVRSSNLKF | 22 |
| GLSIVMPVGGQSSFY | 21 | DAMWGPSSDPAWQRN | 22 |
| IPAEFLENFVRSSNL | 21 | SSFYADWYQPACGKA | 22 |
| SGLSIVMPVGGQSSF | 21 | DIKVQFQSGGNGSPA | 22.5 |
| DYNGWDINTPAFEWY | 21.5 | GAAAAVTLPGLIGLA | 22.5 |
| GYKAADMWGPSSDPA | 21.5 | LSVIMPVGGQSSFYA | 22.5 |
| PAEFLENFVRSSNLK | 21.5 | PSQGMGPSLIGLAMG | 23 |
| QSGLSIVMPVGGQSS | 21.5 | SGLSVIMPVGGQSSF | 23.5 |
| TGSAAIGLSMAGSSA | 21.5 | TPSELGGANMPAEFL | 23.5 |
| NLKFQDAYNAAGGHN | 21.65 | GLSVIMPVGGQSSFY | 24 |
| AEFLENFVRSSNLKF | 22 | GTPSELGGANMPAEF | 24 |
| RPGLPVEYLQVPSPS | 22.5 | YNGWDINTPAFEWYY | 24 |
| SMAGSSAMILAAYHP | 22.5 | QSGLSVIMPVGGQSS | 24.5 |
| TYKWETFLTSELPQW | 22.5 | VTLPGLIGLAGGAAT | 24.5 |
| AAYHPQQFIYAGSLS | 23 | NFNANGTHSWEYWGA | 25 |
| MIGTAAAVVLPGLVG | 23 | WYQPACGKAGCSTYK | 25 |
| NAAGGHNAVFNFPPN | 23 | NMPAEFLENFVRSSN | 25.5 |
| PSQGMGPSLIGLAMG | 23 | SVIMPVGGQSSFYAD | 25.5 |
| QSGGNNSPAVYLLDG | 23 | KRTGSAAVGISMSGS | 26 |
| SIVMPVGGQSSFYSD | 23 | AAAAVTLPGLIGLAG | 26.5 |
| GGYKAADMWGPSSDP | 23.5 | AFSRPGLPVEYLQVP | 26.5 |
| YKWETFLTSELPQWL | 23.5 | AGCSTYKWETFLTSE | 26.5 |
| SNLKFQDAYNAAGGH | 24 | ANMPAEFLENFVRSS | 26.5 |
| WVYCGNGTPNELGGA | 24 | YAGSLSALLDPSQGM | 26.5 |
| YNGWDINTPAFEWYY | 24 | NGTPSELGGANMPAE | 27 |
| AGGYKAADMWGPSSD | 25 | SMSGSSAMILAVNHP | 27 |
| IGTAAAVVLPGLVGL | 25 | SQGMGPSLIGLAMGD | 27 |
| NIPAEFLENFVRSSN | 25 | TSELPSYLASNKGVK | 27 |
| GTAAAVVLPGLVGLA | 25.5 | AGGYKADAMWGPSSD | 27.5 |
| GTPNELGGANIPAEF | 25.5 | PSELGGANMPAEFLE | 27.5 |
| TPNELGGANIPAEFL | 25.5 | RTGSAAVGISMSGSS | 27.5 |
| ADMWGPSSDPAWERN | 26 | AQDDYNGWDINTPAF | 28 |
| QSSFYSDWYSPACGK | 26 | AMWGPSSDPAWQRND | 28.5 |
| AFSRPGLPVEYLQVP | 26.5 | NLKFQDAYNGAGGHN | 28.5 |
| AGCQTYKWETFLTSE | 26.5 | SELGGANMPAEFLEN | 28.5 |
| AMILAAYHPQQFIYA | 26.5 | WETFLTSELPSYLAS | 28.5 |
| ANIPAEFLENFVRSS | 26.5 | HNTRLWVYCGNGTPS | 29 |
| YAGSLSALLDPSQGM | 26.5 | KAGCSTYKWETFLTS | 29 |
| SQGMGPSLIGLAMGD | 27 | KWETFLTSELPSYLA | 29 |
| WYSPACGKAGCQTYK | 27 | SDPAWQRNDPSLHIP | 29 |
| GRDIKVQFQSGGNNS | 27.5 | QSSFYADWYQPACGK | 29.5 |
| SGGNNSPAVYLLDGL | 27.5 | SELPSYLASNKGVKR | 29.5 |
| AQDDYNGWDINTPAF | 28 | ETFLTSELPSYLASN | 30 |
| EYWGAQLNAMKGDLQ | 28 | EFLENFVRSSNLKFQ | 30.5 |
| GQSSFYSDWYSPACG | 28 | FNANGTHSWEYWGAQ | 30.5 |
| MILAAYHPQQFIYAG | 28 | GQSSFYADWYQPACG | 30.5 |
| NGTPNELGGANIPAE | 28 | LTSELPSYLASNKGV | 30.5 |
| NNTRLWVYCGNGTPN | 28 | DPAWQRNDPSLHIPE | 31.5 |
| KAGCQTYKWETFLTS | 28.5 | ELPSYLASNKGVKRT | 31.5 |
| KIRAWGRRLMIGTAA | 28.5 | EYLQVPSAGMGRDIK | 31.5 |
| TAAAVVLPGLVGLAG | 29 | FLTSELPSYLASNKG | 31.5 |
| KPTGSAAIGLSMAGS | 29.5 | KPDLQGTLGASPGGG | 31.5 |
| LAAYHPQQFIYAGSL | 29.5 | NGWDINTPAFEWYYQ | 31.5 |
| AVVLPGLVGLAGGAA | 30 | NGAGGHNAVFNFNAN | 32 |
| PTGSAAIGLSMAGSS | 30 | PAWQRNDPSLHIPEL | 32 |
| EFLENFVRSSNLKFQ | 30.5 | RDIKVQFQSGGNGSP | 32 |
| PNELGGANIPAEFLE | 30.5 | SSDPAWQRNDPSLHI | 32 |
| MAGSSAMILAAYHPQ | 31 | VYCGNGTPSELGGAN | 32 |
| NGWDINTPAFEWYYQ | 31.5 | GNGTPSELGGANMPA | 32.5 |
| NELGGANIPAEFLEN | 32.5 | MKPDLQGTLGASPGG | 32.5 |
| LSALLDPSQGMGPSL | 33 | LSALLDPSQGMGPSL | 33 |
| SRPGLPVEYLQVPSP | 33 | TFLTSELPSYLASNK | 33.5 |
| SSNLKFQDAYNAAGG | 33 | KVRAWGRRLLVGAAA | 34 |
| YQSGLSIVMPVGGQS | 33 | PDLQGTLGASPGGGG | 34 |
| DAGGYKAADMWGPSS | 33.5 | YQPACGKAGCSTYKW | 34 |
| IVMPVGGQSSFYSDW | 33.5 | AGGHNAVFNFNANGT | 34.5 |
| ILAAYHPQQFIYAGS | 34 | DAGGYKADAMWGPSS | 34.5 |
| NNSPAVYLLDGLRAQ | 34 | GSPAVYLLDGLRAQD | 34.5 |
| ETFLTSELPQWLSAN | 34.5 | NGSPAVYLLDGLRAQ | 34.5 |
| NSPAVYLLDGLRAQD | 34.5 | PAVYLLDGLRAQDDY | 34.5 |
| PAVYLLDGLRAQDDY | 34.5 | SPAVYLLDGLRAQDD | 34.5 |
| SPAVYLLDGLRAQDD | 34.5 | AVYLLDGLRAQDDYN | 35 |
| AVYLLDGLRAQDDYN | 35 | PSSDPAWQRNDPSLH | 35 |
| DMWGPSSDPAWERND | 35 | RAQDDYNGWDINTPA | 35 |
| RAQDDYNGWDINTPA | 35 | AMKPDLQGTLGASPG | 35.5 |
| AAAVVLPGLVGLAGG | 35.5 | MSGSSAMILAVNHPD | 35.5 |
| SAMILAAYHPQQFIY | 35.5 | VIMPVGGQSSFYADW | 35.5 |
| FLENFVRSSNLKFQD | 36 | YLASNKGVKRTGSAA | 35.5 |
| GNGTPNELGGANIPA | 36 | AWQRNDPSLHIPELV | 36 |
| TFLTSELPQWLSANR | 36 | FLENFVRSSNLKFQD | 36 |
| YWGAQLNAMKGDLQS | 36 | CGNGTPSELGGANMP | 36.5 |
| GDAGGYKAADMWGPS | 36.5 | SNLKFQDAYNGAGGH | 36.5 |
| NFPPNGTHSWEYWGA | 36.5 | YQSGLSVIMPVGGQS | 36.5 |
| RSSNLKFQDAYNAAG | 36.5 | SLSALLDPSQGMGPS | 37 |
| SSAMILAAYHPQQFI | 36.5 | YCGNGTPSELGGANM | 37 |
| GSSAMILAAYHPQQF | 37 | LPSYLASNKGVKRTG | 38 |
| MGDAGGYKAADMWGP | 37 | ELGGANMPAEFLENF | 38.5 |
| SLSALLDPSQGMGPS | 37 | GHNTRLWVYCGNGTP | 38.5 |
| AAVVLPGLVGLAGGA | 37.5 | AAAVTLPGLIGLAGG | 39 |
| PTQQIPKLVANNTRL | 38 | AVTLPGLIGLAGGAA | 39 |
| ANNTRLWVYCGNGTP | 39 | GAGGHNAVFNFNANG | 39 |
| VYCGNGTPNELGGAN | 39 | WGAQLNAMKPDLQGT | 39.5 |
| VMPVGGQSSFYSDWY | 39.5 | AGSLSALLDPSQGMG | 41 |
| YSPACGKAGCQTYKW | 39.5 | FSRPGLPVEYLQVPS | 41 |
| KWETFLTSELPQWLS | 40 | GSLSALLDPSQGMGP | 41 |
| NDPTQQIPKLVANNT | 40 | IMPVGGQSSFYADWY | 41.5 |
| WETFLTSELPQWLSA | 40 | PSYLASNKGVKRTGS | 42 |
| CGNGTPNELGGANIP | 40.5 | WQRNDPSLHIPELVG | 42.5 |
| RNDPTQQIPKLVANN | 40.5 | YLQVPSAGMGRDIKV | 42.5 |
| TQQIPKLVANNTRLW | 40.5 | AAVTLPGLIGLAGGA | 43 |
| AGSLSALLDPSQGMG | 41 | GAQLNAMKPDLQGTL | 43 |
| FSRPGLPVEYLQVPS | 41 | GDAGGYKADAMWGPS | 44.5 |
| GSLSALLDPSQGMGP | 41 | MGDAGGYKADAMWGP | 45 |
| LQVPSPSMGRDIKVQ | 41 | MWGPSSDPAWQRNDP | 45 |
| AGSSAMILAAYHPQQ | 42 | INTPAFEWYYQSGLS | 45.5 |
| MGRDIKVQFQSGGNN | 42.5 | LGGANMPAEFLENFV | 45.5 |
| VRSSNLKFQDAYNAA | 42.5 | WGPSSDPAWQRNDPS | 45.5 |
| ERNDPTQQIPKLVAN | 43 | AVNHPDQFIYAGSLS | 46.5 |
| QQIPKLVANNTRLWV | 43 | AQLNAMKPDLQGTLG | 47 |
| DPTQQIPKLVANNTR | 44 | GGQSSFYADWYQPAC | 47 |
| ELGGANIPAEFLENF | 44.5 | GRDIKVQFQSGGNGS | 48.5 |
| INTPAFEWYYQSGLS | 45.5 | RNDPSLHIPELVGHN | 48.5 |
| WERNDPTQQIPKLVA | 45.5 | GWDINTPAFEWYYQS | 49 |
| AWERNDPTQQIPKLV | 46 | SGSSAMILAVNHPDQ | 49 |
| GGQSSFYSDWYSPAC | 46 | VYLLDGLRAQDDYNG | 50 |
| MWGPSSDPAWERNDP | 47 |  |  |
| QIPKLVANNTRLWVY | 47 |  |  |
| LAMGDAGGYKAADMW | 47.5 |  |  |
| YCGNGTPNELGGANI | 48.5 |  |  |
| GWDINTPAFEWYYQS | 49 |  |  |
| PAWERNDPTQQIPKL | 49 |  |  |
| SDPAWERNDPTQQIP | 49.5 |  |  |
| DPAWERNDPTQQIPK | 50 |  |  |
| GLAMGDAGGYKAADM | 50 |  |  |
| LIGLAMGDAGGYKAA | 50 |  |  |
| VYLLDGLRAQDDYNG | 50 |  |  |

| **Supplementary Table 8: Predicted Immunogenic CD4 T cell epitopes in ID91 antigens and *M. avium* homologs** | | | | | | |
| --- | --- | --- | --- | --- | --- | --- |
| **Antigen** | **Mtb** | | | ***M. avium*** | | |
|  | **Peptide** | **Combined score** | **Immunogenicity score** | **Peptide** | **Combined score** | **Immunogenicity score** |
| **Mtb Rv3619** | **FQVIYEQANAHGQKV** | 42.89172 | 66.7293 | **FQVIYEQANAHGQKV** | 42.89172 | 66.7293 |
|  | **DAHGAMIRAQAGSLE** | 45.58752 | 70.4688 | **DAHGALIRAQAASLE** | 43.54028 | 86.3507 |
|  | **QGFITQLGRNFQVIY** | 47.99936 | 97.4984 | **QEFITQLGRNFQVIY** | 48.11104 | 97.7776 |
| **Mtb Rv2389** | VVATMFVALLGLSTI | 48.14724 | 96.3681 | IKQIINGLIQAAVPR | 43.42892 | 84.5723 |
|  |  |  |  | GIKQIINGLIQAAVP | 45.64108 | 90.1027 |
| **Mtb Rv3478** | SSAGLMAAAASPYVA | 43.62624 | 94.0656 | LLMSLIATNILGQNT | 38.26292 | 76.1573 |
|  | TELMTLTATNLLGQN | 46.75184 | 82.3796 | ASASMVAAAAPYVVW | 41.4142 | 88.8355 |
|  | AAYETAYRLTVPPPV | 47.63272 | 96.5818 | AGDADLRFMPRLTVV | 42.32116 | 68.3029 |
|  |  |  |  | PPPVIAANRALLMSL | 43.9218 | 90.3045 |
|  |  |  |  | AANRALLMSLIATNI | 45.60804 | 91.5201 |
|  |  |  |  | QDAAAMYMYAGASAA | 46.95108 | 84.3777 |
|  |  |  |  | NQIKSLMPAASAASS | 49.12104 | 95.8026 |
| **Mtb Rv1886** | **QQFIYAGSLSALLDP** | 43.89784 | 98.0446 | **DQFIYAGSLSALLDP** | 42.63972 | 94.5993 |
|  | **AEFLENFVRSSNLKF** | 44.4266 | 96.9665 | **LPVEYLQVPSAGMGR** | 44.41412 | 87.0353 |
|  | **NFVRSSNLKFQDAYN** | 45.72056 | 97.8014 | **AEFLENFVRSSNLKF** | 44.4266 | 96.9665 |
|  | **PAFEWYYQSGLSIVM** | 46.96244 | 94.9061 | **NFVRSSNLKFQDAYN** | 45.72056 | 97.8014 |
|  | **LPVEYLQVPSPSMGR** | 49.7282 | 92.8205 | SAMILAVNHPDQFIY | 47.38332 | 85.4583 |
|  | QIPKLVANNTRLWVY | 49.8748 | 93.187 | AAVGISMSGSSAMIL | 49.16224 | 89.9056 |
|  |  |  |  | **PAFEWYYQSGLSVIM** | 49.41556 | 96.5389 |
| **Bolded** peptides are > 90% similar between Mtb and *M. avium*. | | | | | | |

**Supplementary Figure 3**

**
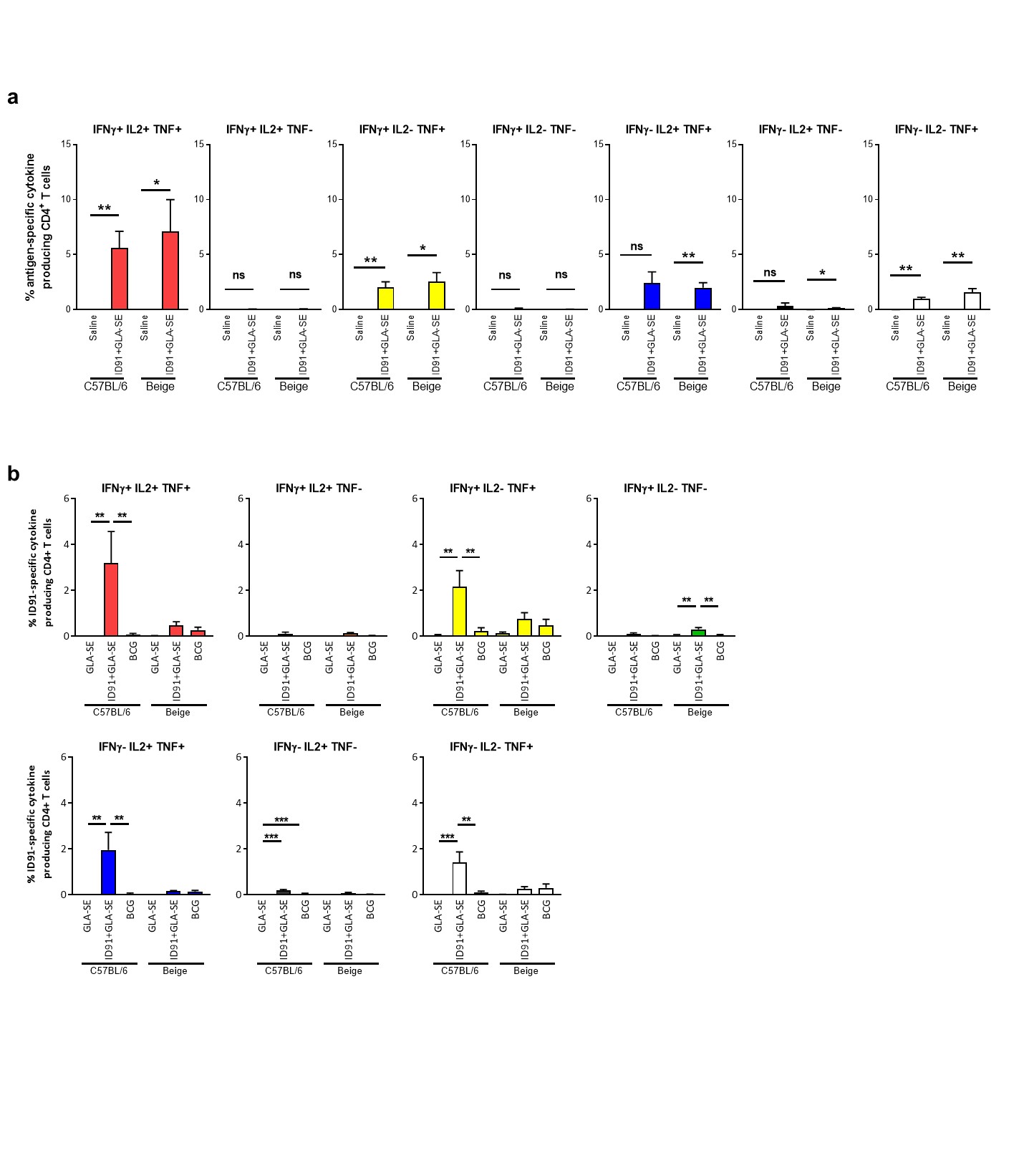
**

**Supplementary Figure 4**

**
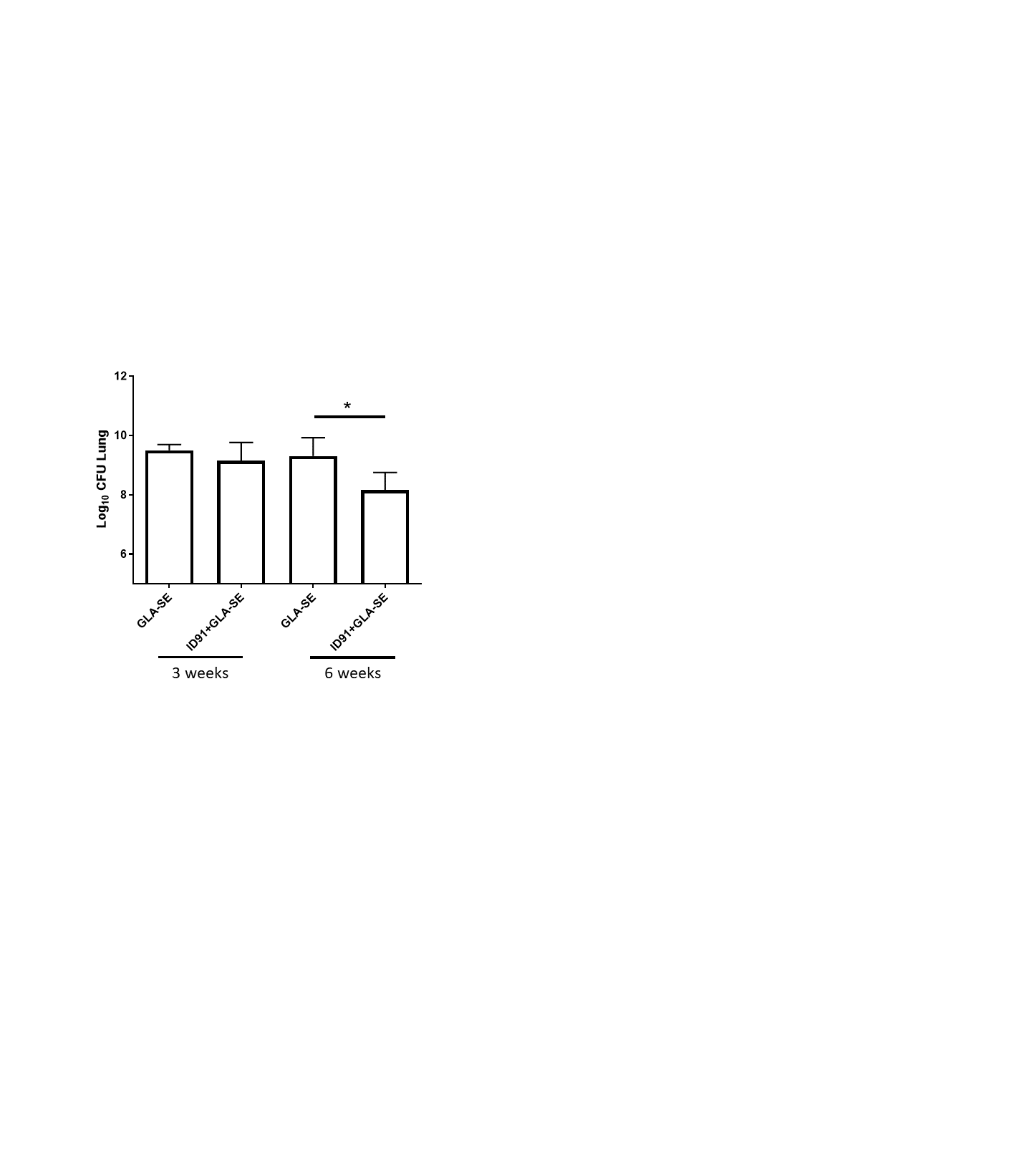
**
